# Genome-scale reconstruction of the metabolic network in Staphylococcus aureus N315: an initial draft to the two-dimensional annotation

**DOI:** 10.1186/1471-2180-5-8

**Published:** 2005-03-07

**Authors:** Scott A Becker, Bernhard Ø Palsson

**Affiliations:** 1 Department of Bioengineering, University of California, San Diego, La Jolla, USA

## Abstract

**Background:**

Several strains of bacteria have sequenced and annotated genomes, which have been used in conjunction with biochemical and physiological data to reconstruct genome-scale metabolic networks. Such reconstruction amounts to a two-dimensional annotation of the genome. These networks have been analyzed with a constraint-based formalism and a variety of biologically meaningful results have emerged. *Staphylococcus aureus *is a pathogenic bacterium that has evolved resistance to many antibiotics, representing a significant health care concern. We present the first manually curated elementally and charge balanced genome-scale reconstruction and model of *S. aureus*' metabolic networks and compute some of its properties.

**Results:**

We reconstructed a genome-scale metabolic network of *S. aureus *strain N315. This reconstruction, termed *i*SB619, consists of 619 genes that catalyze 640 metabolic reactions. For 91% of the reactions, open reading frames are explicitly linked to proteins and to the reaction. All but three of the metabolic reactions are both charge and elementally balanced. The reaction list is the most complete to date for this pathogen. When the capabilities of the reconstructed network were analyzed in the context of maximal growth, we formed hypotheses regarding growth requirements, the efficiency of growth on different carbon sources, and potential drug targets. These hypotheses can be tested experimentally and the data gathered can be used to improve subsequent versions of the reconstruction.

**Conclusion:**

iSB619 represents comprehensive biochemically and genetically structured information about the metabolism of S. aureus to date. The reconstructed metabolic network can be used to predict cellular phenotypes and thus advance our understanding of a troublesome pathogen.

## Background

*Staphylococcus aureus *is a pathogenic gram-positive bacterium that causes a variety of disease conditions, some life-threatening, both in hospital settings and in the community at large. Moreover, various strains of this organism have evolved resistance to some of the most clinically useful antibiotics, including methicillin and vancomycin[1]. Although the mechanisms of antibiotic resistance and infection have been elucidated, there is little published information regarding the basic and systemic biochemical function of *S. aureus*, especially under carefully controlled environmental conditions in chemically-defined media. While some research has been performed towards this goal[2], its scope and extent does not compare to the research undertaken for better-studied model organisms. In fact, the annotated genome sequence of a strain of *S. aureus *contains much more readily-available specific information regarding the organism's metabolism than does a compilation of literature data[3].

The annotated genome of a microorganism, in conjunction with biochemical and physiological data, can be used to reconstruct the metabolic network of that organism[4,5]. Such reconstructed networks consist of a set of chemical reactions that together comprise the known metabolic transformations that take place in a particular organism. These networks are at the genome-scale when all or most of the genes with known metabolic function are included in the network reconstruction. These network reconstructions convey the interactions between cellular components identified from the sequence annotation, and thus reconstructions can be thought of as two-dimensional annotation of the genome[6].

Genome-scale reconstructions (GENREs) represent a biochemically and genetically structured database that can be queried and interrogated using *in silico *analytical methods[7]. With the imposition of appropriate constraints on the reactions in the GENRE, including their exact stoichiometry and reversibility, a genome-scale model (GEM) is formulated. GEMs reflect allowable network states, or phenotypes of a cell, by defining a range of permissible solutions consistent with its mathematical representation[5]. This range of allowable states can be searched for the 'best' growth rates using linear programming methods, and the results from such computations are close to experimental observations [8-11].

GEMs using the constraint-based modeling formalism have been constructed for a number of microorganisms, including *Escherichia coli*[12], *Saccharomyces cerevisiae*[13,14], *Methylobacterium extorquens *[15], *Mannheimia succiniciproducens*[16], *Helicobacter pylori*[17] and *Haemophilus influenzae*[18], and reconstructed networks for human cells are beginning to appear[19]. GEMs are amenable to a wide variety of analysis techniques, yielding a number of interesting results, as recently reviewed[7]. Importantly, GENREs are two-dimensional annotations, are portable, and can be used for computations by different laboratories. In particular, the GENREs for *E. coli *and *S. cerevisiae *have been analyzed by groups around the world (see  for a partial list). These analyses have led to several publications of general interest that focus on such diverse topics as the causes of enzyme dispensability[20], the reconfiguration of metabolism following the loss of a gene or enzyme function[21], and the distribution of metabolic fluxes in microorganisms[22].

GEMs do not only give valuable computational results, but they also provide a wealth of hypotheses that can be experimentally tested[8,23-26]. The generation of easily testable hypothesis by biological models permits model validation and improvement through iterative model building[23]. When the predictions made by a model do not agree with experimental observations, the knowledge that went into the model construction is clearly not complete regarding the area of disagreement. Importantly, an *in silico *model allows us to identify areas where our understanding of an organism is inadequate and where additional experimentation is needed[27].

The work reported herein describes the first manually curated genome-scale elementally and charge balanced metabolic reconstruction and model for the important pathogen *S. aureus*, termed *i*SB619 following a previously described naming convention[25], representing the first draft of its two-dimensional annotation[6]. This GENRE allows for the formulation of hypothesis ranging from relative growth capabilities on different media to the outcome of potential gene deletion experiments. Importantly, due to the curation and refinement necessary to form a functional GEM for *S. aureus*, the work reported contains the most comprehensive metabolic reaction list available for this significant pathogen that is consistent with known phenotypic functions.

## Results and discussion

### Basic network properties

We have formulated a GENRE for *S. aureus *strain N315 consisting of 619 genes, 537 proteins, 640 reactions, and 571 metabolites. The entire reaction list of this GENRE is included in the supplemental material [see Additional file 5 and Additional file 2] and is also available at . A set of metabolic maps graphically representing the GENRE is also available at the same web address. This GENRE was built without the benefit of an earlier manually curated reconstruction and model and should be considered a first-draft to the two-dimensional annotation of the *S. aureus *genome. Unlike other initial GENREs[17,18,28], the *S. aureus *GENRE is nearly completely elementally and charge balanced. All but three reactions produce no net change in terms of chemical elements and charge. Of these three reactions, one (1,4-dihydroxy-2-naphthoate octaprenyltransferase, abbreviated DHNAOT) is never used because it is a dead-end (see below), one (phosphatidic acid synthase, abbreviated PASYN_SA) is a weighted combination of various fatty acids to form an average phosphatidate molecule for this organism, and one (2,3-diketo-5-methylthio-1-phosphopentane degradation reaction, abbreviated DKMPPD2) participates in methionine and spermidine metabolism.

Most of the reactions (91%) in the GENRE are associated with one or more genes with only 9% (59) of the reactions included without a known gene. The reactions that do not have a gene association are principally transport reactions, allowing metabolites to cross the cell membrane, and reactions that involve the formation of lipids and other cell wall components [see Additional file 6]. The inclusion of these reactions, as well as the reactions which we were able to associate with a gene despite their absence in the genome annotation (detailed in Materials and Methods), is a legacy-data based enhancement of genome-annotation based knowledge on the metabolism of *S. aureus*. We provide the most comprehensive reaction list to date, including reactions that were added based on systemic analysis [see Additional file 9] – the GEM cannot produce biomass without them. All reactions that are associated with genes are also associated with proteins, and they are represented by what have been termed gene-protein-reaction (GPR) associations[25], which are available as Boolean statements connecting genes to reactions in the supplementary material [see Additional file 8] and in graphical form at . Basic properties of the reconstructed network are summarized in Table 1.

Particularly because this is a first-pass reconstruction, the network has a significant number of dead-end metabolites, as other GENREs do[14,17,18,25]. These dead-ends are compounds that are either only produced or only consumed by reactions in the network. Three hypotheses exist regarding the presence of a dead-end metabolite in a reconstructed network: (1) other enzymes required to produce or consume the metabolite may be missing in the reconstruction, (2) the reaction that causes the dead-end may have been misidentified based on a homology search and may not actually occur in the organism, or (3) the dead-end may exist in organism. Because the accumulation or depletion of any compound cannot occur in a steady-state, any reaction in the network involving any of these compounds cannot be used in a computed network state. In total, 108 reactions present in the reconstruction involve dead-end metabolites. All of these reactions have an associated gene and are included because of genetic evidence that they are present in *S. aureus*. Subsequent additions to the model will likely close some of these gaps. These reactions are further detailed in the supplemental material [see Additional file 3].

A reaction representing biomass formation, consisting of 58 metabolites required for cellular growth, has been defined and is detailed comprehensively in the supplemental material [see Additional file 1]. Key components of this reaction include amino acids, nucleotides, lipids, and cell wall constituents. Because data describing the biomass composition of *S. aureus *could not be located in the literature, data from *Bacillus subtilis *was substituted where necessary[29]; quantitative data specific to *S. aureus *accounts for only a small fraction of the biomass function. Although a comprehensive biomass function has been published for *E. coli *and used to analyze the networks of other initial reconstructions[17,18], this was not appropriate for *S. aureus *because of the differences between gram-positive and gram-negative bacteria. The relative quantities of each required metabolite were included in the biomass function when information existed, but many of the trace compounds were included in small ratios that are not quantitatively accurate. It has been shown previously that the calculated biomass production is relatively insensitive to the exact ratios used in the biomass function[30]. The biomass function is a key element of the linear programming (LP) formulation used for hypothesis generation because it allows for the computational prediction of growth.

### Minimal media and growth requirements

Linear programming using the assumptions of flux-balance analysis (FBA) allows for the computation of feasible steady-state fluxes through a reaction network that maximize a particular objective and satisfy various constraints, including stoichiometry, thermodynamics, and enzyme capacity[7,31-33]. Specifically, we used FBA to determine fluxes leading to optimal growth subject to constraints on the usage of each reaction. This principle allowed us to systematically predict a minimal media composition capable of supporting growth of *S. aureus*. A literature search revealed experimental growth requirements[3,34,35] for *S. aureus *and they are compared with the computational predictions (Table 2). These requirements are for growth with oxygen, nitrate, or nitrite as a terminal electron acceptor. Computationally, we predict that *S. aureus *can grow with a variety of carbon sources, and Table 2 presents a glucose minimal medium because the available experimental data assumes that glucose is the carbon source. This table should be considered as a prediction of the growth requirements of *S. aureus *derived from its GENRE. Although some of the components in the medium seem obvious, like phosphate and a carbon source, they still serve as validation for the GEM. If we were to computationally predict that growth was possible in the absence of a carbon source, it would quickly become apparent that something was amiss with the GEM. An agreement between the computationally-predicted and the experimentally-determined requirements indicates areas where simple model predictions are consistent with existing experimental data.

The primary difference between the computationally predicted growth requirements and those from experimental data is the amino acid requirement. Kuroda et. al[3] report that the six amino acids listed in Table 2 are specifically required for strain N315 to grow and speculate that, since the strain has pathways for the synthesis of all amino acids, regulation might require the presence of these amino acids. *i*SB619 does not account for regulatory effects and as such predicts media requirements as if regulatory processes allow any gene to be expressed at the needed levels. The transcriptional regulatory network reduces the functionality of a constraint-based metabolic model by limiting which reactions are active at a given time[36]. If enzymes required for the synthesis of a given amino acid are encoded in the genome, but are not sufficiently transcribed or translated due to regulatory processes, the cell will require that amino acid as a component of the medium even though a purely metabolic model indicates otherwise.

We experimentally predict growth if any one of the nine amino acids or derivatives listed in the table is provided. Each of these compounds can either provide nitrogen through a deamination reaction or be directly converted into another compound that can provide nitrogen. *S. aureus *is said to require an organic source of nitrogen provided by amino acids[37], so the requirement of at least one amino acid is not surprising. It should be noted that the data from Kuroda et al[3] listing six essential amino acids is "unpublished data" and thus this information should be viewed with some skepticism. Furthermore, *S. aureus *has shown the ability to grow without amino acids previously thought to be required[37]; thus the amino acid requirement appears to be flexible. The differences in computationally-predicted and experimentally-determined essential amino acids highlight an area that has been historically under considered.

**Figure 1:**
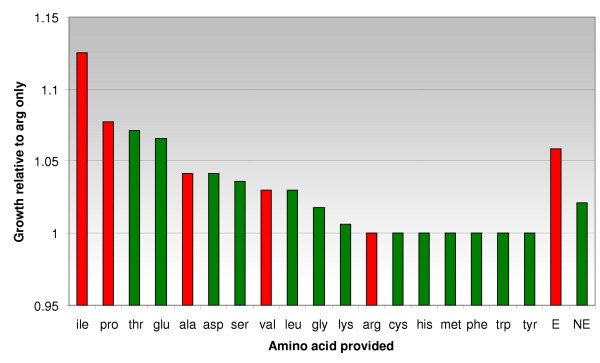
**Amino acid contributions to growth. **The results of adding equivalent quantities of each amino acid to the media are shown here. Red bars represent amino acids that are reported essential in the literature, and green bars are amino acids that are not. Arginine is present in all cases and is the baseline against which the rest of the values are normalized. On average, adding an essential amino acid to the media allows better growth than does adding a non-essential amino acid. E stands for average essential amino acid, and NE stands for average non-essential amino acid. Two amino acids do not have transporters in the genome annotation and are not included here for that reason.

To explore the discrepancy between experimental results and computational predictions, we elected to study *in silico *the effect of adding each amino acid individually to the predicted minimal media listed (with arginine present at all times). The individual results are shown in Figure 1. We found that, on average, providing one of amino acids noted as essential from experimental data led to more biomass production than providing one amino acid not listed as essential. Using a Wilcoxon rank sum test with Matlab (The MathWorks, Inc., Natick, MA), these results are only 5.6% likely to be the result of random chance. The relative biomass production that we predict should be taken as a hypothesis that can be tested experimentally. It is not unreasonable that a pathogen would have developed regulation that leads it to require the uptake of amino acids that significantly aid its growth, especially when they are readily available in its typical environment. In essence, we predict that *S. aureus *can grow more efficiently by uptaking certain amino acids rather than synthesizing them, even though its genome encodes that functionality. Although one might intuitively think that this would be the case for all amino acids, the results in Figure 1 indicate that the synthesis of some amino acids is not predicted to substantially inhibit growth under the conditions studied (cys, his, met, phe, trp, tyr).

### Deletion Study

In order to determine the effects of the deletion of a reaction from the network, as would occur in a gene knock-out experiment, FBA is used with the additional constraint that the flux through a particular reaction is zero. This allows for the rapid prediction of the results of gene deletions and also reaction deletions, as occur when a selective enzyme inhibitor is used. We calculated the effects of all single reaction and gene deletions both on minimal medium and on a rich media (consisting of all amino acids, nucleotides, and protoheme). We found that, on rich media, 130 reaction deletions and 88 gene deletions are computationally predicted to be lethal. On minimal media, 230 reaction deletions and 168 gene deletions are predicted to be lethal. These predictions are detailed in the supplemental material [see Additional file 4 and Additional file 7]. There are fewer lethal gene deletions than reaction deletions because some essential reactions are not associated with genes, some essential reactions are associated with isozymes, and some genes catalyze multiple reactions. When analyzed in the context of GPR associations, we found that 20 (9%) of the reactions that are essential on minimal media are associated with isozymes. This calculation indicates that gene dispensability is explained by the presence of isozymes less often than in *S. cerevisiae *(14.6%-27.8%) [20]. We note that the distinction between isozymes that independently catalyze a reaction and multiple gene products required simultaneously for a reaction is not always clear from the data sources used in this reconstruction.

We took the results of the reaction deletion study on rich media and searched PubMed for chemical inhibitors of each of those reactions (Table 3). Most of the inhibitors listed do not have any published information regarding their effectiveness in *S. aureus *to the best of our knowledge. These computational predictions can be experimentally tested with targeted gene deletions or the inhibitors listed. Due to the diversity of biological systems in which the inhibitors were initially discovered, gene deletions would be expected to agree with our predictions more than the use of chemical inhibitors. There are a variety of reasons why any one of these inhibitors listed may have no utility whatsoever as a drug. For example, there may be no way for the inhibitor to actually enter a cell. Nevertheless, when considered as an initial guess at potential drugs, Table 3 represents hypotheses formed by a systems-level approach that has not been applied to an organism as clinically troublesome as *S. aureus *before.

Additionally, we considered comparing the results of our gene deletion study with the results of an existing experimental approach to determine gene essentiality. Unfortunately, although we are aware of two large-scale studies on gene essentiality in *S. aureus*[38,39], there does not exist a comprehensive, publicly-available resource listing all essential genes in this organism. There is a comprehensive gene essentiality study for the related organism *B. subtilis*[40], but this is a different organism. Should comprehensive gene essentiality data become available for *S. aureus*, a comparison between experimental data and the predictions detailed herein can easily serve as validation for this model and identify problem areas. The absence of a comprehensive, publicly-available data set regarding gene essentiality in this organism is in itself powerful motivation to undertake the reconstruction detailed in this paper, as the reconstruction can rapidly predict essential metabolic genes that can later be screened experimentally as potential drug targets.

### Growth Phenotypes

**Figure 2:**
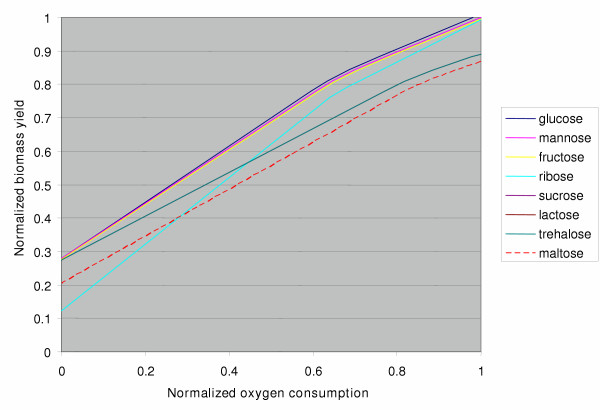
**Relative growth efficiency with different carbon sources. **The in silico growth of *i*SB619 varies depending on which carbon source is provided and the amount of oxygen present. The predicted efficiency of carbon incorporation into biomass is shown here as a function on the oxygen consumption. Growth rate is normalized relative to the number of carbon atoms per molecule. Oxygen consumption is normalized relative to optimal oxygen consumption for each carbon source. Trehalose, lactose, and sucrose all overlap (the trehalose line indicates all three). The legend is presented in the same order as the carbon sources appear in the figure, top to bottom.

We computed the sensitivity of growth rate to oxygen uptake on a variety of different carbon sources (Figure 2). The carbon source uptake rate is restricted to the same molar maximum for all calculations. As expected, biomass production increases with oxygen uptake up to the point where there is no longer an oxygen limitation. In addition, the carbon sources which contain more carbon atoms also generally allow greater biomass production. For example, trehalose, which contains twice as much carbon as glucose, allows close to twice as much biomass production as an equivalent molar amount of glucose. The normalization used, explained in the figure caption, allows a quick analysis of the efficiency of the network in utilizing different carbon sources. Importantly, these predictions can be tested experimentally as a means of validating and improving the current GEM.

## Conclusion

The work reported is the first genome-scale metabolic reconstruction for the pathogenic bacterium *S. aureus *and represents the first draft to its two-dimensional genome annotation. The GENRE with the GPR associations represents a chemically and genetically structured database derived from the underlying data. When the properties of this GENRE are analyzed using FBA, the model computationally predicts phenotypic states. Based on the surprising paucity of physiological growth data available for this organism, especially under carefully defined conditions, the predictions made by the GEM are best viewed as hypotheses. These hypotheses can be experimentally tested, with similar results serving as validation for the GEM and dissimilar results describing failure modes of the GEM. These failure modes can be more interesting than correct predictions because they provide direction for improvement of the GEM and point to areas in metabolism and regulation that need further investigation. The analysis of failure modes and subsequent improvement of the GEM constitute a cycle of iterative model building[23,27], with the potential to significantly improve a GEM. Second generation models of *E. coli *and *S. cerevisiae *have made substantial improvements over initial genome-scale reconstructions. These enhancements include greater coverage of metabolism, explicit GPR associations, more detailed enzyme localization, and better enforcement of elemental and charge balancing. We expect that future enhancements to *i*SB619 will improve the accuracy of the reaction network and the GPR associations.

The scope of hypotheses that can be devised with a constraint-based metabolic model ranges from the relatively mundane, such as prediction of relative growth on different carbon sources, to the potentially groundbreaking, such as the determination of novel prospective drug targets. With an organism as harmful as *S. aureus*, any advancement of knowledge is welcome.

## Methods

### Reconstruction of the metabolic network

A genome annotation for *S. aureus *strain N315 was downloaded from the Comprehensive Microbial Resource (CMR) at The Institute for Genomic Research (TIGR) website[41] and used to form a gene index. Each key metabolic pathway present in map form on the Kyoto Encyclopedia of Genes and Genomes (KEGG) website[42] was then examined to extract reactions that genomic data suggest occur in this strain. These reactions were matched to proteins and genes based on the information provided by both TIGR and KEGG. After this pathway-by-pathway approach, the predicted functionality of each gene in the genome was examined manually, both in the TIGR annotation and on the KEGG website to find additional metabolic reactions that are not present in any KEGG map. Inconsistencies between TIGR and KEGG were handled on a case-by-case basis to determine what functionality should be assigned to a given gene. For example, in a case where one source (either TIGR or KEGG) indicated that the gene was a "conserved hypothetical protein" but the other source listed a specific metabolic function, the gene was generally given the specific function. If both sources gave conflicting functions, a reaction was included in the model if it was present in related organisms but was only associated with a gene that had conflicting annotations if another suitable gene without conflicting information could not be found.

After assembling the network based on genomic data, missing functions were noted based on physiological data regarding this organism, as well as *B. subtilis *and *E. coli*. Two books proved particularly helpful for this process[37,43]. Likely reactions were added to the model based on pathways present in related organisms. For example, some cell-wall components known to be in *S. aureus *could not be produced without the addition of reactions in various pathways present in *B. subtilis*. Membrane-bound transporters were added whenever evidence existed that a metabolite could enter and/or exit the cell. For example, if data indicated that a given carbon source could be used, a transporter was added to the reconstruction. Potential genes for some of these reactions were located by best-hit BLAST analysis against *E. coli *and *B. subtilis*. Each reaction without a gene association was checked against the genome annotations for *E. coli *and *B. subtilis *to determine if a gene exists in either organism for a given function. A homology search was used with any gene located in *E. coli *or *B. subtilis *against the entire *S. aureus *N315 genome. A reaction was putatively associated with a gene based on the E value provided by BLAST and the annotation information for that gene from both KEGG and TIGR. If a *S. aureus *gene did not have any functional information included in either annotation, a BLAST E value of 0.05 was considered sufficient to make an association. If functional information was present and differed from the specific reaction under consideration, a better E value would be necessary; the precise E value required was a judgment call and depended on the specificity of the annotation information. An association based on a relatively large E value should not be considered as a claim that a gene product catalyzes a reaction, but a suggestion as to a candidate gene [see Additional file 9]. Transporters were not associated with genes unless an annotation indicated with some degree of specificity that the gene had the given function. Whenever possible, the reactions in the network were balanced elementally and with respect to charge, where compound charge and molecular formula were based on a cellular pH of 7.2. The end result can be visualized as a stoichiometric matrix **S **with each column representing a reaction and each row a metabolite, with each element representing the stoichiometric coefficient. This reconstruction process, including accounting for GPR associations, and the subsequent analysis described below were done using the software program SimPheny™ (Genomatica Inc., San Diego, CA).

### Biomass composition

Because no thorough biomass composition has been published for *S. aureus*, the relative production of metabolites required for growth was taken to be similar to that published for the related gram-positive organism *B. subtilis*[29]. The fatty acid composition of the lipids required for growth was, however, based on data specific to *S. aureus*[44]. Small amounts of a number of minor biomass constituents were added to the biomass function in equal amounts to account for their necessity in cellular growth. Further details are provided in the supplemental material [see Additional file 1].

### Computation of phenotypic states and deletion study

With the reconstructed metabolic network and biomass function defined, flux-balance analysis (FBA) [31] was used to find optimal growth phenotypes. Briefly, linear programming was used to find a complete set of metabolic fluxes (**v**) that are consistent with all constraints, namely steady-state network operation (eq. 1 below) and reaction rate limitations (eq. 2 below), and which maximize the production of biomass components in the defined ratio. This corresponds to the following linear programming problem:

max Z = v_growth_

Subject to

**S **• **v **= 0     (1)

α_i _< v_i _< β_i _    (2)

where **S **is the stoichiometric matrix described above, and α_i _and β_i _define the minimum and maximum allowable fluxes through each reaction v_i_. The flux range was set arbitrarily high for all internal reactions so that no internal reaction restricted the network, with the exception of reactions known to be irreversible, which have a minimum flux of zero. The inputs to the system were restricted where necessary (for example, limiting the amount of glucose available to the cell). The function v_growth _is a special reaction taking as substrates all biomass metabolites, ATP and water, and producing ADP, protons, and phosphate (as a result of the non-growth associated ATP maintenance requirement). SimPheny™ (Genomatica Inc, San Diego) was used for all FBA calculations.

The value of Z computed with the above procedure can either be zero or greater than zero depending on the inputs and outputs that are allowed, corresponding to the nutrients provided in the media. A zero value is a computational prediction of no growth; this commonly occurs when an essential nutrient like a carbon source is not provided. Any value greater than zero corresponds to cellular growth.

For the deletion study, each reaction was individually constrained to have zero flux and maximal biomass production was computed. Reactions were considered essential if no biomass could be produced without their usage. Gene deletions were computed in a similar manner, but all reactions requiring the presence of a given gene were simultaneously restricted to zero flux prior to computing maximal biomass production.

### Minimal media

The minimal media was determined computationally with the systematic testing of distinct inputs. In short, different combinations of molecules were allowed to enter the reaction network until the minimal group that allowed biomass production, or non-zero Z, was found. Importantly, the minimal media computed here does not discriminate between extremely slow, inefficient growth and rapid growth; it is only concerned that some amount of biomass production is calculated.

## List of abbreviations

CMR: Comprehensive Microbial Resource

EC: Enzyme Commission

FBA: flux-balance analysis

GEM: genome-scale model

GENRE: genome-scale reconstruction

GPR: gene-protein-reaction

KEGG: Kyoto Encyclopedia of Genes and Genomes

LP: linear programming

TIGR: The Institute for Genomic Research

## Authors' contributions

SAB carried out all aspects of the work and drafted the manuscript. BOP conceived of the study, participated in its design and coordination, and helped draft the manuscript. All authors read and approved the final manuscript.

## Supplementary Material

Additional File 1 Cellular biomass demand table This is a detailed, quantitative listing of the macromolecules required for cellular growth. Click here for file

Additional File 2 Compound abbreviations This is a listing of the compound abbreviations used in the reconstruction and the corresponding formal compound names. Click here for file

Additional File 3 Dead-end reactions This is a listing of the reactions that the current version of the model will never use because they involve a dead-end metabolite. Click here for file

Additional File 4 Lethal reaction deletions on minimal media This is a listing of all of the reactions that are predicted to be essential for growth on minimal media and their corresponding gene associations. Click here for file

Additional File 5 Network reaction list This is a comprehensive list of all of the reactions that are in the model. Click here for file

Additional File 6 Reactions without any gene association This is a list of the reactions that are included in the model without any gene association and rationale for their inclusion. Click here for file

Additional File 7 Lethal reaction deletions on rich media This is a listing of all of the reactions that are predicted to be essential for growth on rich media and their corresponding gene associations. Click here for file

Additional File 8 Boolean gene-reaction associations This is a listing of reaction abbreviations along with the genes that are required for those reactions, in a Boolean form. Click here for file

Additional File 9 Reactions that were added based on systemic evidence This is a list of the reactions that were added to the reconstruction based on systemic analysis (they do not appear in an obvious fashion in the genome annotation), and the gene(s) with which they are associated in cases where a gene could be located based on homology searches. Click here for file

## Figures and Tables

**Table 1:** Basic network properties

Genes	619
Proteins	537
Reactions	640
Reactions with gene associations	581
Metabolites	571
Exchange fluxes	84

**Table 2:** Computational and experimental minimal media

**Computational**	**Experimental**	
proline OR arginine OR glutamate OR alanine OR	alanine	amino acids
aspartate OR glycine OR ornithine OR serine OR	glycine	
threonine	isoleucine	
	arginine	
	valine	
	proline	
cytidine OR cytosine OR uridine OR uracil		nucleotides

phosphate	phosphate	cofactors, ions, etc.
sulfate	sulfate	
nicotinamide OR nicotinate	nicotinamide OR nicotinate	
iron	iron? (disagreement in literature)	
oxygen OR ((nitrate OR nitrite) AND protoheme)	oxygen assumed	
thiamin	thiamin	
	
	biotin	
	calcium pantothenate	
	ammonium	
glucose	glucose	carbon source

The computed and experimentally determined minimal media for growth of *S. aureus *compare reasonably well. The most noticeable difference is the amino acid requirement, which can be attributed to regulation, as detailed in the text. The boolean statements (AND/OR) are standard; for example, there are three terminal electron acceptors that can be members of the computational minimal medium, o2, no3, and no2, but both no3 and no2 also require the presence of pheme. The absence of an explicit logic statement between lines is equivalent to using AND; for example, all 6 amino acids listed are required together in the experimental minimal medium.

**Table 3:** Essential enzymes and potential chemical inhibitors

**Enzyme name**	**Potential Inhibitor**	**Prior testing?**	**Reference**
acetyl-CoA carboxylase	pseudopeptide pyrrolidine dione antibiotics	SA, B	[45]
4-amino-4-deoxychorismate synthase	(6s)-6-fluoroshikimate	B	[46]
Adenosylmethionine decarboxylase	CGP 40215A, AdoMao	B	[47,48]
asparagine synthase (glutamine-hydrolysing)	mucochloric and mucobromic acids, L-cysteine sulfinic acid	B	[49,50]
dihydrofolate reductase	methylpteridines	B	[51]
dihydropteroate synthase	Sulfone and sulfanilamide sulfa drugs	B	[52,53]
3-dehydroquinate synthase	carbocyclic inhibitors	B	[54]
FMN adenylyltransferase (FAD synthase)	Riboflavin 5'-pyrophosphate	F	[55]
glycerol-3-phosphate dehydrogenase (NADP)	5-n-alk(en)ylresorcinols	NF	[56]
glutamine synthetase	L-methionine sulfoximine, aminomethylene-bisphosphonic acid derivatives	NF	[57,58]
glutamyl-tRNA reductase	see table I in paper	NF	[59]
GTP cyclohydrolase I	Diamino-6-hydroxypyrimidine, pterins	NF	[60,61]
Hydroxymethylglutaryl CoA reductase (ir)	statins	NF	[62,63]
Hydroxymethylglutaryl CoA synthase (ir)	beta-lactone, 3-Hydroxy-3-methylglutaryldithio-coenzyme A	NF	[64,65]
isopentenyl-diphosphate D-isomerase	NE21650	NF	[66]
methionine adenosyltransferase	adduncts 14 and 16	B	[67]
Phosphatidate phosphatase	Propranolol	NF	[68]
phosphoribosylpyrophosphate synthetase	MRPP, ARPP	NF	[69]
riboflavin synthase	9-D-ribitylamino-1,3,7,9-tetrahydro-2,6,8-purinetriones	B	[70]
spermidine synthase	adenosylspermidine, dicyclohexylamine	SA, B	[71,72]
thiamine transport via ABC system	azidobenzoyl derivatives of thiamin, methylene blue	F	[73,74]
thioredoxin reductase	Arsenicals, Aurothioglucose	NF	[75,76]
UDP-N-acetylglucosamine 4-epimerase	uridine analogs	NF	[77]
UDP-N-acetylenolpyruvoylglucosamine reductase	4-thiazolidinones	B	[78]

A reasonable number of enzymes that are computationally predicted to be essential for the growth of *S. aureus *have inhibitors. These molecules are potential drugs against this organism. The prior testing column uses abbreviations to indicate if we located evidence that the listed compounds had been tested in *S. aureus *(SA), other bacteria (B), fungi (F), or if no evidence was located (NF). The interested reader should consult the relevant references for full details regarding these potential inhibitors.
