# Supplementary material for: Genome-scale reconstruction of the metabolic network in Staphylococcus aureus N315: an initial draft to the two-dimensional annotation
Source: BMC Microbiol. 2005 Mar 7;5:8. doi: 10.1186/1471-2180-5-8 (PMC1079855; doi:10.1186/1471-2180-5-8)
Supplement: Additional File 1 — Cellular biomass demand table This is a detailed, quantitative listing of the macromolecules required for cellular growth. [file 1471-2180-5-8-S1.pdf]

Overview

**Component**	**% of Biomass**
Protein	60%
RNA	12%
DNA	3%
Lipids	5%
Lipoteichoic Acid	2%
Cell Wall Components	15%
Trace Components	3%

Full Description

**Quantity**	**Compound Abbreviation**	**Compound Name**
0.01	10fthf	10-Formyltetrahydrofolate (C20H21N7O7)
0.42	12dgr_SA	1,2-Daicylglycerol (Saureus) (C1560H3065O42)
1.27	26dap-LL	LL-2,6-Diaminoheptanedioate (C7H14N2O4)
0.01	SA_FREE_FA	SA Free Fatty Acids (X)
0.38	acgam	N-Acetyl-D-glucosamine (C8H15NO6)
1.22	acgam1p	N-Acetyl-D-glucosamine 1-phosphate (C8H14NO9P)
6.6	ala-L	L-Alanine (C3H7NO2)
3.11	amp	AMP (C10H12N5O7P)
3.88	arg-L	L-Arginine (C6H15N4O2)
4.48	asn-L	L-Asparagine (C4H8N2O3)
4.48	asp-L	L-Aspartate (C4H6NO4)
20000	atp	ATP (C10H12N5O13P3)
2.51	clpn_SA	Cardiolipin (Saureus) (C3270H6330O434P100)
2.2	cmp	CMP (C9H12N3O8P)
0.79	cys-L	L-Cysteine (C3H7NO2S)
0.76	damp	dAMP (C10H12N5O6P)
0.53	dcmp	dCMP (C9H12N3O7P)
0.012068	dgdcg_SA2	Diglucosyl-diacylglycerol (SA) 2 (C2160H4165O542)
0.6	dgmp	dGMP (C10H12N5O7P)
0.73	dtmp	dTMP (C10H13N2O8P)
0.01	fad	FAD (C27H31N9O15P2)
0.23	gam1p	D-Glucosamine 1-phosphate (C6H13NO8P)
5.3	glcp_SA	Glucosyl Phosphoglycerol (SA) (C996H1868N62O1116P150)
8.72	gln-L	L-Glutamine (C5H10N2O3)
9.68	glu-L	L-Glutamate (C5H8NO4)
3.51	gly	Glycine (C2H5NO2)
4.01	gmp	GMP (C10H12N5O8P)
20000	h2o	H2O (H2O)
0.01	hemeO	Heme O (C49H56FeN4O5)
1.45	his-L	L-Histidine (C6H9N3O2)
4.06	ile-L	L-Isoleucine (C6H13NO2)
5.21	leu-L	L-Leucine (C6H13NO2)
5.45	lys-L	L-Lysine (C6H15N2O2)
1.94	met-L	L-Methionine (C5H11NO2S)
0.01	mlthf	5,10-Methylenetetrahydrofolate (C20H21N7O6)
0.01	nad	Nicotinamide adenine dinucleotide (C21H26N7O14P2)
0.01	nadp	Nicotinamide adenine dinucleotide phosphate (C21H25N7O17P3)
0.01	pala_SA2	Phosphatidylalanine_SA2 (C1710H3315N50O242P50)
0.89	pe_SA	Phosphatidylethanolamine (Saureus) (C1660H3365N50O192P50)
2.31	pg_SA	Phosphatidylglycerol (Saureus) (C1710H3365O292P50)
0.01	pgly_SA2	Phosphatidylglycine_SA2 (C1660H3215N50O242P50)
3.33	phe-L	L-Phenylalanine (C9H11NO2)
0.01	pleu_SA2	Phosphatidylleucine_SA2 (C1860H3615N50O242P50)
0.15	plys_SA2	Phosphatidyllysine_SA2 (C1860H3715N100O242P50)
2.12	pro-L	L-Proline (C5H9NO2)
0.01	ptrc	Putrescine (C4H14N2)
2.6	ser-L	L-Serine (C3H7NO3)
0.01	sheme	Siroheme (C42H36FeN4O16)
0.01	spmd	Spermidine (C7H22N3)
2.86	tcam	minor teichoic acid (acetylgalactosamine glucose phosphate, n=30) (C420H750N30O420P30)
0.01	thf	5,6,7,8-Tetrahydrofolate (C19H21N7O6)
0.01	thm	Thiamin (C12H17N4OS)
2.54	thr-L	L-Threonine (C4H9NO3)
1.27	trp-L	L-Tryptophan (C11H12N2O2)
2.3	tyr-L	L-Tyrosine (C9H11NO3)
1.78	uamr	UDP-N-acetylmuramate (C20H28N3O19P2)
2.39	ump	UMP (C9H11N2O9P)
4.12	val-L	L-Valine (C5H11NO2)
