# Supplementary material for: Genome-scale reconstruction of the metabolic network in Staphylococcus aureus N315: an initial draft to the two-dimensional annotation
Source: BMC Microbiol. 2005 Mar 7;5:8. doi: 10.1186/1471-2180-5-8 (PMC1079855; doi:10.1186/1471-2180-5-8)
Supplement: Additional File 2 — Compound abbreviations This is a listing of the compound abbreviations used in the reconstruction and the corresponding formal compound names. [file 1471-2180-5-8-S2.pdf]

Compound Abbreviation	Compound Name
10fthf	10-Formyltetrahydrofolate
12dag3p	1,2-Diacyl-sn-glycerol 3-phosphate
12dgr_EC	1,2-Diacylglycerol (E.coli)
12dgr_SA	1,2-Daicylglycerol (Saureus)
13dpg	3-Phospho-D-glyceroyl phosphate
15dap	1,5-Diaminopentane
1pyr5c	1-Pyrroline-5-carboxylate
23ddhb	2,3-Dihydro-2,3-dihydroxybenzoate
23dhdp	2,3-Dihydrodipicolinate
23dhmb	(R)-2,3-Dihydroxy-3-methylbutanoate
23dhmp	(R)-2,3-Dihydroxy-3-methylpentanoate
25aics	(S)-2-[5-Amino-1-(5-phospho-D-ribosyl)imidazole-4-carboxamido]succinate
25dhpp	2,5-Diamino-6-hydroxy-4-(5'-phosphoribosylamino)-pyrimidine
26dap-LL	LL-2,6-Diaminoheptanedioate
26dap-M	meso-2,6-Diaminoheptanedioate
2a3pp	2-Amino-3-phosphonopropanoate
2ahbut	(S)-2-Aceto-2-hydroxybutanoate
2aobut	L-2-Amino-3-oxobutanoate
2cpr5p	1-(2-Carboxyphenylamino)-1-deoxy-D-ribulose 5-phosphate
2dda7p	2-Dehydro-3-deoxy-D-arabino-heptonate 7-phosphate
2dhp	2-Dehydropantoate
2dmmq8	2-Demethylmenaquinone 8
2dmmql8	2-Demethylmenaquinol 8
2dr1p	2-Deoxy-D-ribose 1-phosphate
2dr5p	2-Deoxy-D-ribose 5-phosphate
2hymeph	2-(Hydroxymethyl)phenol
2ippm	2-Isopropylmaleate
2kmb	2-keto-4-methylthiobutyrate
2mahmp	2-Methyl-4-amino-5-hydroxymethylpyrimidine diphosphate
2mbcoa	2-Methylbutanoyl-CoA
2mbdhl	S-(2-Methylbutanoyl)-dihydrolipoamide
2me4p	2-C-methyl-D-erythritol 4-phosphate
2mpdhl	S-(2-Methylpropanoyl)-dihydrolipoamide
2obut	2-Oxobutanoate
2ombz	2-Octaprenyl-6-methoxy-1,4-benzoquinone
2ommb	2-Octaprenyl-3-methyl-6-methoxy-1,4-benzoquinone
2p4c2me	2-phospho-4-(cytidine 5'-diphospho)-2-C-methyl-D-erythritol
2pg	D-Glycerate 2-phosphate
2pglyc	2-Phosphoglycolate
2shchc	2-Succinyl-6-hydroxy-2,4-cyclohexadiene-1-carboxylate
34hpp	3-(4-Hydroxyphenyl)pyruvate
3AStmyn	3''-Adenylylstreptomycin
3AStrmyn	3''-Adenylylspectinomycin
3R3haACP	(3R)-3-Hydroxyacyl-[acyl-carrier protein]
3c2hmp	3-Carboxy-2-hydroxy-4-methylpentanoate
3c3hmp	3-Carboxy-3-hydroxy-4-methylpentanoate
3c4mop	3-Carboxy-4-methyl-2-oxopentanoate
3dhq	3-Dehydroquinate
3dhsk	3-Dehydroshikimate
3g12dgr_SA	3-D-Glucosyl-1,2-diacylglycerol
3g12dgr_SA2	3-D-Glucosyl-1,2-diacylglycerol
3hbcoa	(S)-3-Hydroxybutyryl-CoA
3hdcoa	(S)-3-Hydroxydecanoyl-CoA
3hddcoa	(S)-3-Hydroxydodecanoyl-CoA
3hhcoa	(S)-3-Hydroxyhexanoyl-CoA
3hhdcoa	(S)-3-Hydroxyhexadecanoyl-CoA
3hmrsACP	R-3-hydroxy-myristoyl-ACP
3hocoa	(S)-3-Hydroxyoctanoyl-CoA
3htdcoa	(S)-3-Hydroxytetradecanoyl-CoA
3ig3p	C'-(3-Indolyl)-glycerol 3-phosphate
3mbdhl	S-(3-Methylbutanoyl)-dihydrolipoamide
3mob	3-Methyl-2-oxobutanoate
3mop	(S)-3-Methyl-2-oxopentanoate
3odcoa	3-Oxodecanoyl-CoA
3oddcoa	3-Oxododecanoyl-CoA
3ohcoa	3-Oxohexanoyl-CoA
3ohdcoa	3-Oxohexadecanoyl-CoA
3oocoa	3-Oxooctanoyl-CoA
3otdcoa	3-Oxotetradecanoyl-CoA
3oxoACP_delete	3-Oxoacyl-[acyl-carrier protein]
3pg	3-Phospho-D-glycerate
3php	3-Phosphohydroxypyruvate
3pop	3-Phosphonopyruvate
3psme	5-O-(1-Carboxyvinyl)-3-phosphoshikimate
4abut	4-Aminobutanoate
4abz	4-Aminobenzoate

4adcho	4-amino-4-deoxychorismate
4ampm	4-Amino-2-methyl-5-phosphomethylpyrimidine
4c2me	4-(cytidine 5'-diphospho)-2-C-methyl-D-erythritol
4izp	4-Imidazolone-5-propanoate
4mhetz	4-Methyl-5-(2-hydroxyethyl)-thiazole
4mop	4-Methyl-2-oxopentanoate
4mpetz	4-Methyl-5-(2-phosphoethyl)-thiazole
4pasp	4-Phospho-L-aspartate
4ppan	D-4'-Phosphopantothenate
4ppcys	N-((R)-4-Phosphopantothenoyl)-L-cysteine
4r5au	4-(1-D-Ribitylamino)-5-aminouracil
5aizc	5-amino-1-(5-phospho-D-ribosyl)imidazole-4-carboxylate
5aop	5-Amino-4-oxopentanoate
5aprbu	5-Amino-6-(5'-phosphoribitylamino)uracil
5apru	5-Amino-6-(5'-phosphoribosylamino)uracil
5caiz	5-phosphoribosyl-5-carboxyaminoimidazole
5dpmev	(R)-5-Diphosphomevalonate
5mdr1p	5-Methylthio-5-deoxy-D-ribose 1-phosphate
5mdru1p	5-Methylthio-5-deoxy-D-ribulose 1-phosphate
5mta	5-Methylthioadenosine
5mthf	5-Methyltetrahydrofolate
5mtr	5-Methylthio-D-ribose
5pmev	(R)-5-Phosphomevalonate
6hmhpt	6-hydroxymethyl dihydropterin
6hmhptpp	6-hydroxymethyl-dihydropterin pyrophosphate
6pgc	6-Phospho-D-gluconate
6pgl	6-phospho-D-glucono-1,5-lactone
6pthp	6-Pyruvoyl-5,6,7,8-tetrahydropterin
8aonn	8-Amino-7-oxononanoate
ACP	acyl carrier protein
Cit-Mg	Citrate-Mg
DGDG_SA	Diglucosyl Diglyceride (SA)
Lac6p	Lactose-6-phosphate
SA_FREE_FA	SA Free Fatty Acids
Sptmyn	Spectinomycin
Stmyn	Streptomycin
aacoa	Acetoacetyl-CoA
ac	Acetate
acACP	Acetyl-ACP
acald	Acetaldehyde
accoa	Acetyl-CoA
acg5p	N-Acetyl-L-glutamyl 5-phosphate
acg5sa	N-Acetyl-L-glutamate 5-semialdehyde
acgam	N-Acetyl-D-glucosamine
acgam1p	N-Acetyl-D-glucosamine 1-phosphate
acgam6p	N-Acetyl-D-glucosamine 6-phosphate
acglu	N-Acetyl-L-glutamate
achms	O-Acetyl-L-homoserine
acmalt	Acetyl-maltose
acmam	N-Acetyl-D-muramoate
acmama	N-Acetyl-D-muramoyl-L-alanine
acmana	N-Acetyl-D-mannosamine
acmanap	N-Acetyl-D-mannosamine 6-phosphate
acnam	N-Acetylneuraminate
acorn	N2-Acetyl-L-ornithine
acser	O-Acetyl-L-serine
actACP	Acetoacetyl-ACP
actn-R	(R)-Acetoin
actp	Acetyl phosphate
adcobdam	Adenosyl cobyrinate diamide
adcobhex	adenosyl-cobyric acid
ade	Adenine
adn	Adenosine
adp	ADP
adpglc	ADPglucose
adphep-D,D	ADP-D-glycero-D-manno-heptose
adphep-L,D	ADP-L-glycero-D-manno-heptose
adprib	ADPribose
agm	Agmatine
ahcys	S-Adenosyl-L-homocysteine
ahdt	2-Amino-4-hydroxy-6-(erythro-1,2,3-trihydroxypropyl)dihydropteridine triphosphate
ahdt_#1 [deleted 07/14/2004 02:2	2-Amino-4-hydroxy-6-(erythro-1,2,3-trihydroxypropyl)dihydropteridine triphosphate
aicar	5-Amino-1-(5-Phospho-D-ribosyl)imidazole-4-carboxamide
air	5-amino-1-(5-phospho-D-ribosyl)imidazole
akg	2-Oxoglutarate
ala-B	beta-Alanine
ala-D	D-Alanine

ala-L	L-Alanine
alaala	D-Alanyl-D-alanine
alac-S	(S)-2-Acetolactate
alatrna	L-Alanyl-tRNA(Ala)
alpro	S-Aminomethyldihydrolipoylprotein
amet	S-Adenosyl-L-methionine
ametam	S-Adenosylmethioninamine
amob	S-Adenosyl-4-methylthio-2-oxobutanoate
amp	AMP
anth	Anthranilate
apoACP	apoprotein [acyl carrier protein]
aps	Adenosine 5'-phosphosulfate
ara5p	D-Arabinose 5-phosphate
arg-L	L-Arginine
argsuc	N(omega)-(L-Arginino)succinate
argtrna	L-Arginyl-tRNA(Arg)
asn-L	L-Asparagine
asntrna	L-Asparaginyl-tRNA(Asn)
asp-L	L-Aspartate
aspsa	L-Aspartate 4-semialdehyde
asptrna	L-Aspartyl-tRNA(Asp)
atp	ATP
b2coa	trans-But-2-enoyl-CoA
betald	Betaine aldehyde
bgly	N-Benzoylglycine
btamp	Biotinyl-5'-AMP
btcoa	Butanoyl-CoA
btn	Biotin
bz	Benzoate
cbasp	N-Carbamoyl-L-aspartate
cbp	Carbamoyl phosphate
cdp	CDP
cdpdag	CDPdiacylglycerol
cdpdag_EC	CDPdiacylglycerol (E coli)
cdpdag_SA	CDPdiacylglycerol (Saureus)
cdpea	CDPethanolamine
cdpglyc	CDPglycerol
chol	Choline
chor	Chorismate
cit	Citrate
citr-L	L-Citrulline
ckdo	CMP-3-deoxy-D-manno-octulosonate
clpn_EC	Cardiolipin (Ecoli)
clpn_SA	Cardiolipin (Saureus)
cmp	CMP
co2	CO2
coa	Coenzyme A
cobalt2	Co2+
cpppg1	Coproporphyrinogen I
cpppg3	Coproporphyrinogen III
crn	L-Carnitine
csn	Cytosine
ctp	CTP
cu2	Cu2+
cys-L	L-Cysteine
cyst-L	L-Cystathionine
cystrna	L-Cysteinyl-tRNA(Cys)
cytd	Cytidine
dad-2	Deoxyadenosine
dadp	dADP
damp	dAMP
dann	7,8-Diaminononanoate
datp	dATP
db4p	3,4-dihydroxy-2-butanone 4-phosphate
dc2coa	trans-Dec-2-enoyl-CoA
dcacoa	Decanoyl-CoA (n-C10:0CoA)
dcamp	N6-(1,2-Dicarboxyethyl)-AMP
dcdp	dCDP
dcmp	dCMP
dctp	dCTP
dd2coa	trans-Dodec-2-enoyl-CoA
ddcaACP	Dodecanoyl-ACP (n-C12:0ACP)
ddcacoa	Dodecanoyl-CoA (n-C12:0CoA)
decdp	all-trans-Decaprenyl diphosphate
dgal6p	D-Galactose 6-phosphate
dgdcg_SA	Diglucosyl-diacylglycerol (SA)
dgdcg_SA2	Diglucosyl-diacylglycerol (SA) 2

dgdp	dGDP
dgmp	dGMP
dgtp	dGTP
dha	Dihydroxyacetone
dhap	Dihydroxyacetone phosphate
dhf	7,8-Dihydrofolate
dhlam	Dihydrolipoamide
dhlpro	Dihydrolipolprotein
dhna	1,4-Dihydroxy-2-naphthoate
dhnpt	2-Amino-4-hydroxy-6-(D-erythro-1,2,3-trihydroxypropyl)-7,8-dihydropteridine
dhor-S	(S)-Dihydroorotate
dhpmp	Dihydroneopterin monophosphate
dhpt	Dihydropteroate
dkmpp	2,3-diketo-5-methylthio-1-phosphopentane
dmlz	6,7-Dimethyl-8-(1-D-ribityl)lumazine
dmpp	Dimethylallyl diphosphate
dnad	Deamino-NAD+
dpcoa	Dephospho-CoA
drib	Deoxyribose
dscl	dihydrosirohydrochlorin
dtbt	Dethiobiotin
dtdp	dTDP
dtmp	dTMP
dttp	dTTP
dudp	dUDP
dump	dUMP
duri	Deoxyuridine
dutp	dUTP
e4p	D-Erythrose 4-phosphate
eig3p	D-erythro-1-(Imidazol-4-yl)glycerol 3-phosphate
etha	Ethanolamine
etoh	Ethanol
f1p	D-Fructose 1-phosphate
f6p	D-Fructose 6-phosphate
fa1	Fatty acid (Iso-C14:0)
fa10	Fatty acid (Anteiso-C17:1)
fa11	Fatty acid (Iso-C17:0)
fa12	Fatty acid (Anteiso-C17:0)
fa19a	Fatty Acid (Anteiso-C19:0)
fa20n	Fatty Acid (C20:0)
fa3	Fatty acid (Iso-C15:0)
fa4	Fatty acid (Anteiso-C15:0)
fa5	Fatty acid (Iso-C16:1)
fa6	Fatty acid (iso-C16:0)
fa9	Fatty acid (Iso-C17:1)
fad	FAD
fadh2	FADH2
fald	Formaldehyde
fdp	D-Fructose 1,6-bisphosphate
fdxox	Oxidized ferredoxin
fdxrd	Reduced ferredoxin
fe2	Fe2+
fgam	N2-Formyl-N1-(5-phospho-D-ribosyl)glycinamide
fmn	FMN
fmnh2	Reduced FMN
for	Formate
forglu	N-Formimidoyl-L-glutamate
fpram	2-(Formamido)-N1-(5-phospho-D-ribosyl)acetamidine
fprica	5-Formamido-1-(5-phospho-D-ribosyl)imidazole-4-carboxamide
frdp	Farnesyl diphosphate
frmd	Formamide
fru	D-Fructose
fum	Fumarate
g1p	D-Glucose 1-phosphate
g3p	Glyceraldehyde 3-phosphate
g3pc	sn-Glycero-3-phosphocholine
g3pe	sn-Glycero-3-phosphoethanolamine
g3pg	Glycerophosphoglycerol
g3pi	sn-Glycero-3-phospho-1-inositol
g3ps	Glycerophosphoserine
g6p	D-Glucose 6-phosphate
galt1p	Galactitol 1-phosphate
gam1p	D-Glucosamine 1-phosphate
gam6p	D-Glucosamine 6-phosphate
gar	N1-(5-Phospho-D-ribosyl)glycinamide
gcald	Glycolaldehyde
gdp	GDP

gdptp	Guanosine 3'-diphosphate 5'-triphosphate
glc-D	D-Glucose
glcn	D-Gluconate
glcp_SA	Glucosyl Phosphoglycerol (SA)
gln-L	L-Glutamine
glu-D	D-Glutamate
glu-L	L-Glutamate
glu1sa	L-Glutamate 1-semialdehyde
glu5sa	L-Glutamate 5-semialdehyde
glutrna	L-Glutamyl-tRNA(Glu)
glx	Glyoxylate
gly	Glycine
glyald	D-Glyceraldehyde
glyb	Glycine betaine
glyc	Glycerol
glyc-R	(R)-Glycerate
glyc3p	Glycerol 3-phosphate
glyclt	Glycolate
glycogen	glycogen
glytrna	Glycyl-tRNA(Gly)
gmhep17bp	D-Glycero-D-manno-heptose 1,7-bisphosphate
gmhep1p	D-Glycero-D-manno-heptose 1-phosphate
gmhep7p	D-Glycero-D-manno-heptose 7-phosphate
gmp	GMP
grdp	Geranyl diphosphate
gtca1	glycerol teichoic acid (n=25), unlinked, unsubstituted
gtca2	glycerol teichoic acid (n=25), unlinked, D-ala substituted
gtca3	glycerol teichoic acid (n=25), unlinked, glucose substituted
gthox	Oxidized glutathione
gthrd	Reduced glutathione
gtp	GTP
gua	Guanine
h	H+
h2o	H2O
h2o2	Hydrogen peroxide
h2s	Hydrogen sulfide
hco3	Bicarbonate
hcys-L	L-Homocysteine
hdca	Hexadecanoate (n-C16:0)
hdcea	hexadecenoate (n-C16:1)
hdd2coa	trans-Hexadec-2-enoyl-CoA
hdeACP	Hexadecenoyl-ACP (n-C16:1ACP)
hemeA_#1	Heme A
hemeO	Heme O
hepdp	all-trans-Heptaprenyl diphosphate
hexdp	all-trans-Hexaprenyl diphosphate
his-L	L-Histidine
hisp	L-Histidinol phosphate
histd	L-Histidinol
hmbil	Hydroxymethylbilane
hmgcoa	Hydroxymethylglutaryl-CoA
hom-L	L-Homoserine
hpglu	Tetrahydropteroyltri-L-glutamate
hpyr	Hydroxypyruvate
hx2coa	trans-Hex-2-enoyl-CoA
hxan	Hypoxanthine
hxcoa	Hexanoyl-CoA
ibcoa	Isobutyryl-CoA
ichor	Isochorismate
icit	Isocitrate
id3acald	Indole-3-acetaldehyde
idp	IDP
ile-L	L-Isoleucine
iletrna	L-Isoleucyl-tRNA(Ile)
imacp	3-(Imidazol-4-yl)-2-oxopropyl phosphate
imp	IMP
indpyr	Indolepyruvate
inost	myo-Inositol
ipdp	Isopentenyl diphosphate
itp	ITP
ivcoa	Isovaleryl-CoA
k	K+
kdo	3-Deoxy-D-manno-2-octulosonate
kdo2lipid4	KDO(2)-lipid IV(A)
kdo2lipid4L	KDO(2)-lipid IV(A) with laurate
kdo8p	3-Deoxy-D-manno-octulosonate 8-phosphate
kdolipid4	KDO-lipid IV(A)

lac-D	D-Lactate
lac-L	L-Lactate
leu-L	L-Leucine
lipa	KDO(2)-lipid (A)
lipidA	2,3,2'3'-Tetrakis(beta-hydroxymyristoyl)-D-glucosaminyl-1,6-beta-D-glucosamine 1,4'-bisphosphate
lipidAds	Lipid A Disaccharide
lipidX	2,3-Bis(3-hydroxytetradecanoyl)-beta-D-glucosaminyl 1-phosphate
lpam	Lipoamide
lpro	Lipoylprotein
lps_EC	lipopolysaccharide (Ecoli)
lys-L	L-Lysine
lystrna	L-Lysine-tRNA (Lys)
mal-L	L-Malate
malACP	Malonyl-[acyl-carrier protein]
malcoa	Malonyl-CoA
male	Maleate
malm	Maleamate
malt	Maltose
man1p	D-Mannose 1-phosphate
man6p	D-Mannose 6-phosphate
melib	Melibiose
meoh	Methanol
met-L	L-Methionine
methf	5,10-Methenyltetrahydrofolate
mettrna	L-Methionyl-tRNA (Met)
mev-R	(R)-Mevalonate
mg2	Mg
mhpglu	5-Methyltetrahydropteroyltri-L-glutamate
mi1p-D	1D-myo-Inositol 1-phosphate
mlthf	5,10-Methylenetetrahydrofolate
mn2	Mn2+
mnl1p	D-Mannitol 1-phosphate
mobd	Molybdate
mql8	Menaquinol 8
mqn8	Menaquinone 8
myrsACP	Myristoyl-ACP (n-C14:0ACP)
n6all26d	N6-Acetyl-LL-2,6-diaminoheptanedioate
na1	Sodium
nac	Nicotinate
nad	Nicotinamide adenine dinucleotide
nadh	Nicotinamide adenine dinucleotide - reduced
nadp	Nicotinamide adenine dinucleotide phosphate
nadph	Nicotinamide adenine dinucleotide phosphate - reduced
nal2a6o	N-Acetyl-L-2-amino-6-oxopimelate
ncam	Nicotinamide
nh4	Ammonium
nh4oh	Ammonium hydroxide
ni2	Ni2+
nicrnt	Nicotinate D-ribonucleotide
no2	Nitrite
no3	Nitrate
nop	D-Nopaline
o2	O2
o2-	Superoxide anion
oaa	Oxaloacetate
oc2coa	trans-Oct-2-enoyl-CoA
occoa	Octanoyl-CoA (n-C8:0CoA)
ocdca	octadecanoate (n-C18:0)
ocdcea	octadecenoate (n-C18:1)
octdp	all-trans-Octaprenyl diphosphate
octeACP	Octadecenoyl-ACP (n-C18:1ACP)
octp	Octopine
orn	Ornithine
orot	Orotate
orot5p	Orotidine 5'-phosphate
pa_EC	phosphatidate (E.coli)
pa_SA	phosphatidate (Saureus)
pala_SA	Phosphatidylananine_SA
pala_SA2	Phosphatidylalanine_SA2
palmACP	Palmitoyl-ACP (n-C16:0ACP)
pan4p	Pantetheine 4'-phosphate
pant-R	(R)-Pantoate
pap	Adenosine 3',5'-bisphosphate
paps	3'-Phosphoadenylyl sulfate
pe_EC	Phosphatidylethanolamine (ecoli)
pe_SA	Phosphatidylethanolamine (Saureus)
pep	Phosphoenolpyruvate

peptido_EC	Peptidoglycan subunit of Escherichia coli
pg_EC	Phosphatidylglycerol (Ecoli)
pg_HP	Phosphatidylglycerol Hp specific
pg_SA	Phosphatidylglycerol (Saureus)
pgly_SA	Phosphatidylglycine_SA
pgly_SA2	Phosphatidylglycine_SA2
pgp_EC	Phosphatidylglycerophosphate (Ecoli)
pgp_SA	Phosphatidylglycerophosphate (Saureus)
phe-L	L-Phenylalanine
pheme	Protoheme
phom	O-Phospho-L-homoserine
phpyr	Phenylpyruvate
pi	Phosphate
pime	Pimelate
pleu_SA	Phosphatidylleucine_SA
pleu_SA2	Phosphatidylleucine_SA2
plys_SA	Phosphatidyllysine_SA
plys_SA2	Phosphatidyllysine_SA2
pmcoa	Pimeloyl-CoA
pmtcoa	Palmitoyl-CoA (n-C16:0CoA)
pnto-R	(R)-Pantothenate
ppbng	Porphobilinogen
pphn	Prephenate
ppi	Diphosphate
ppp9	Protoporphyrin
pppg9	Protoporphyrinogen IX
pppi	Inorganic triphosphate
pram	5-Phospho-beta-D-ribosylamine
pran	N-(5-Phospho-D-ribosyl)anthranilate
prbamp	1-(5-Phosphoribosyl)-AMP
prbatp	1-(5-Phosphoribosyl)-ATP
prfp	1-(5-Phosphoribosyl)-5-[(5-phosphoribosylamino)methylideneamino]imidazole-4-carboxamide
prlp	5-[(5-phospho-1-deoxyribulos-1-ylamino)methylideneamino]-1-(5-phosphoribosyl)imidazole-4-carboxamide
pro-L	L-Proline
prpp	5-Phospho-alpha-D-ribose 1-diphosphate
ps_EC	phosphatidylserine (Ecoli)
ps_SA	phosphatidylserine (Saureus)
psd5p	Pseudouridine 5'-phosphate
pser-L	O-Phospho-L-serine
ptrc	Putrescine
pyr	Pyruvate
q8	Ubiquinone-8
q8h2	Ubiquinol-8
r1p	alpha-D-Ribose 1-phosphate
r5p	alpha-D-Ribose 5-phosphate
raffin	Raffinose
rbflvrd	Reduced riboflavin
rbl-L	L-Ribulose
rhcys	S-Ribosyl-L-homocysteine
rib-D	D-Ribose
ribflv	Riboflavin
ru5p-D	D-Ribulose 5-phosphate
ru5p-L	L-Ribulose 5-phosphate
s	Sulfur
s7p	Sedoheptulose 7-phosphate
salc6p	Salicin 6-phosphate
sbt-D	D-Sorbitol
sbzcoa	O-Succinylbenzoyl-CoA
scl	sirohydrochlorin
sdhlam	S-Succinyldihydrolipoamide
ser-L	L-Serine
sertrna	L-Seryl-tRNA(Ser)
sheme	Siroheme
skm	Shikimate
skm5p	Shikimate 5-phosphate
sl26da	N-Succinyl-LL-2,6-diaminoheptanedioate
sl2a6o	N-Succinyl-2-L-amino-6-oxoheptanedioate
so3	Sulfite
so4	Sulfate
spmd	Spermidine
sql	Squalene
ssaltpp	Succinate semialdehyde-thiamin diphosphate anion
suc6p	Sucrose 6-phosphate
sucbz	o-Succinylbenzoate
succ	Succinate
succoa	Succinyl-CoA
suchms	O-Succinyl-L-homoserine

sucr	Sucrose
sucsal	Succinic semialdehyde
tag6p-D	D-Tagatose 6-phosphate
tagdp-D	D-Tagatose 1,6-biphosphate
tcam	minor teichoic acid (acetylgalactosamine glucose phosphate, n=30)
td2coa	trans-Tetradec-2-enoyl-CoA
tdcoa	Tetradecanoyl-CoA (n-C14:0CoA)
tdeACP	Tetradecenoyl-ACP (n-C14:1ACP)
thdp	2,3,4,5-Tetrahydrodipicolinate
thf	5,6,7,8-Tetrahydrofolate
thfglu	Tetrahydrofolyl-[Glu](2)
thm	Thiamin
thmmp	Thiamin monophosphate
thmpp	Thiamine diphosphate
thr-L	L-Threonine
thrtrna	L-Threonyl-tRNA(Thr)
thymd	Thymidine
trdox	Oxidized thioredoxin
trdrd	Reduced thioredoxin
tre6p	alpha,alpha'-Trehalose 6-phosphate
trnaala	tRNA(Ala)
trnaarg	tRNA(Arg)
trnaasn	tRNA(Asn)
trnaasp	tRNA(Asp)
trnacys	tRNA(Cys)
trnaglu	tRNA (Glu)
trnagly	tRNA(Gly)
trnaile	tRNA(Ile)
trnalys	tRNA(Lys)
trnamet	tRNA(Met)
trnaser	tRNA(Ser)
trnathr	tRNA(Thr)
trp-L	L-Tryptophan
ttdca	tetradecanoate (n-C14:0)
tyr-L	L-Tyrosine
u23ga	UDP-2,3-bis(3-hydroxytetradecanoyl)glucosamine
u3aga	UDP-3-O-(3-hydroxytetradecanoyl)-N-acetylglucosamine
u3hga	UDP-3-O-(3-hydroxytetradecanoyl)-D-glucosamine
uaagmda	Undecaprenyl-diphospho-N-acetylmuramoyl-(N-acetylglucosamine)-L-ala-D-glu-meso-2,6-diaminopimeloyl-D-ala-D-ala
uaccg	UDP-N-acetyl-3-O-(1-carboxyvinyl)-D-glucosamine
uacgam	UDP-N-acetyl-D-glucosamine
uacmam	UDP-N-acetyl-D-mannosamine
uagmda	Undecaprenyl-diphospho-N-acetylmuramoyl-L-alanyl-D-glutamyl-meso-2,6-diaminopimeloyl-D-alanyl-D-alanine
uama	UDP-N-acetylmuramoyl-L-alanine
uamag	UDP-N-acetylmuramoyl-L-alanyl-D-glutamate
uamr	UDP-N-acetylmuramate
udcp	Undecaprenol
udcpdp	Undecaprenyl diphosphate
udcpp	Undecaprenyl phosphate
udp	UDP
udpacgal	UDP-N-acetyl-D-galactosamine
udpg	UDPglucose
udpgal	UDPgalactose
ugmd	UDP-N-acetylmuramoyl-L-alanyl-D-gamma-glutamyl-meso-2,6-diaminopimelate
ugmda	UDP-N-acetylmuramoyl-L-alanyl-D-glutamyl-meso-2,6-diaminopimeloyl-D-alanyl-D-alanine
ump	UMP
uppg1	Uroporphyrinogen I
uppg3	Uroporphyrinogen III
ura	Uracil
urcan	Urocanate
urea	Urea
uri	Uridine
utp	UTP
val-L	L-Valine
xan	Xanthine
xmp	Xanthosine 5'-phosphate
xu5p-D	D-Xylulose 5-phosphate
zn2	Zinc
