# Supplementary material for: Genome-scale reconstruction of the metabolic network in Staphylococcus aureus N315: an initial draft to the two-dimensional annotation
Source: BMC Microbiol. 2005 Mar 7;5:8. doi: 10.1186/1471-2180-5-8 (PMC1079855; doi:10.1186/1471-2180-5-8)
Supplement: Additional File 3 — Dead-end reactions This is a listing of the reactions that the current version of the model will never use because they involve a dead-end metabolite. [file 1471-2180-5-8-S3.pdf]

Reaction	Reason for Dead End
3M2OBLOXRD	no further conversion for product
3M2OPLOXRD	no further conversion for product
4M2OPLOXRD	no further conversion for product
6PHBG	no further conversion for product
ABTAr	no gene for glutamate decarboxylase to produce substrate
ACLDC	no further conversion for product
ADCYRS	product of pathway not included in model
ADPRDP	further DNA metabolism not included; accounted for differently in biomass
AGMHE	no further conversion for product
AHCYSNS	no further conversion for byproduct (rhcys)
ALATRS	tRNA not extended beyond synthesis
ALCD1	both product and reactant not included in model further
AMAA	substrate and product not further included in model--biotin not required in model now
AMAOTr	no need for biotin in model
ARGTRS	tRNA not extended beyond synthesis
ASNTRS	tRNA not extended beyond synthesis
ASPTRS	tRNA not extended beyond synthesis
BACCL	no need for biotin in model
BTS3R	no need for biotin in model
CDPMEK	present in annotation, but product unused
CHCOAL	biotin pathway left incomplete
CIT-Mgt	transporter included based on kegg annotation and inclusion in b. subtilis
Coabc	annotation includes this function, but no clear use for cobalt inside cell
CRNabc	annotation includes this function, but no clear use for crn inside cell
Cut1	included in annotation but no source of copper inside
CYSTRS	tRNA not extended beyond synthesis
DHNAOT	no way to produce substrate in genome annotation--quinones not considered in biomass
DRBK	deoxyribose not required in biomass
FGLU	no reaction to use formamide located in annotation
FLVR	no reactions using reduced riboflavin located in annotation
FLVR(NAD)	no reactions using reduced riboflavin located in annotation
G3PD7	quinone unused
G6PDH2R	due to gap in pentose phosphate pathway
GALTPTS	in annotation but no use inside cell currently
GCALDD	no use for product in annotation
GLYALDt	potential transporter in annotation, but no use inside cell
GLYAT	no further consumption (or generation) of product
GLYD	no use for product
GLYTRS	tRNA not extended beyond synthesis
GPDDA1	in annotation, but no way to produce substrates
GPDDA2	in annotation, but no way to produce substrates
GPDDA3	in annotation, but no way to produce substrates
GPDDA4	in annotation, but no way to produce substrates
GPDDA5	in annotation, but no way to produce substrates
GTHP	in annotation, but no clear use for compounds
GTPDPK	no clear use for product
HEMEAS	hemeA not included in biomass at this point
HETZK	in annotation, but disconnected from rest of network
HEXTT	quinone unused
HSERTA	product unused elsewhere
HXPRT	substrate not produced in model
ILETRS	tRNA not extended beyond synthesis
INDPYRD	product and reactant not further used in model
KAS10	minor fatty acid componts left out of biomass--so reactions appear to be unused
KAS14	minor fatty acid componts left out of biomass--so reactions appear to be unused
KAS15	minor fatty acid componts left out of biomass--so reactions appear to be unused

KAS16	minor fatty acid componts left out of biomass--so reactions appear to be unused
KAS17	minor fatty acid componts left out of biomass--so reactions appear to be unused
KAS5	minor fatty acid componts left out of biomass--so reactions appear to be unused
KAS7	minor fatty acid componts left out of biomass--so reactions appear to be unused
KAS9	minor fatty acid componts left out of biomass--so reactions appear to be unused
LYSDC	product not further used in model
LYSTRS	tRNA not extended beyond synthesis
MALTAT	product unused elsewhere
MEPCT	present in annotation, but substrate not created
METTRS	tRNA not extended beyond synthesis
MG2abc	common media includes mg2, so transporter added
MHPGLUT	byproducts unused elsewhere
MI1PP	product and reactant not further used in model
MNabc	common media includes MN, so transporter added
MNt2	common media includes MN, so transporter added
MOBDabc	transporter included in annotation but compound not used in model
NADH8	particular quinone unused in model due to not being regenerated
NADH9	particular quinone unused in model due to not being regenerated
NALN6	included in annotation, but disconnected from rest of model
NBAH	bz not used in model
NDPK4	dttp not required in biomass
NIabc	nickel transporter annotated but no function for nickel in model
NOPD	nop not further used
OCTD	octp not further used
OOR2r	ferredoxins not regenerated in this model
ORNTA	byproduct not used elsewhere
PGLYCP	product and reactant not further used in model
PMANM	product not further used in model
PMPK	substrate not produced in model
PTHPS	product not further used in model
PUNP2	product not further used in model
PYRZAM	product and reactant not further used in model
RAFH	product and reactant not further used in model
RBK_L1	product not further used in model
S3AdT	functionality not yet extended--not traditional metabolism
SBTD_Dr	product not further used in model
SERTRS	tRNA not extended beyond synthesis
SHCHCS2	quinone unused
SPODM	superoxide not generated in model
SPT3ADT	functionality not yet extended--not traditional metabolism
SQLS	sql not used in model
THFGLUS	product not further used in model
THRTRS	tRNA not extended beyond synthesis
TMPPP	product unused elsewhere
UAGPT3	product unused elsewhere
UDCPKr	substrate not produced in model
UDPDPS	substrate not produced in model
UDPG4E	product not further used in model
UPPDC2	product not further used in model
URFGTT	product and reactant not further used in model
YUMPS	product not further used in model
ZNABC	transporter in annotation, but no function for zn in model
