# Supplementary material for: Genome-scale reconstruction of the metabolic network in Staphylococcus aureus N315: an initial draft to the two-dimensional annotation
Source: BMC Microbiol. 2005 Mar 7;5:8. doi: 10.1186/1471-2180-5-8 (PMC1079855; doi:10.1186/1471-2180-5-8)
Supplement: Additional File 4 — Lethal reaction deletions on minimal media This is a listing of all of the reactions that are predicted to be essential for growth on minimal media and their corresponding gene associations. [file 1471-2180-5-8-S4.pdf]

Reaction Abbreviation	Gene Association	Isozymes?
ACCOAC	SA1357, SA1358, SA1434, SA1435, SA1522, SA1523	no
ACGK	SA0176	
ACHBS	SA1859, SA1860, SA2008	partial
ACLS	SA1859, SA1860, SA2008	partial
ACOTA	SA0179	
ADCL	SA0670	
ADCS	SA0669	
ADK1	SA2027	
ADKd	SA2027	
ADMDCr		
ADPT	SA1461	
ADSK	SA2456	
ADSL1	SA1724	
ADSL2r	SA1724	
ADSS	SA0016	
AGPR	SA0178	
AICART	SA0925	
AIRC2	SA0917	
AIRC3	SA0916	
AKGDa	SA1245	
AKGDb	SA1244	
ALAR	SA1231, SA1874	yes
ALATA_D	SA1571	
ANPRT	SA1201	
ANS	SA0668, SA1199, SA1200	partial
APRAUR	SA1586, SA1588	no
ASAD	SA1226	
ASNS1	SA0922	
ASPKi	SA1163, SA1225	yes
ASPTA	SA1749	
ATPPRT	SA2471	
BPNT		
CHORM	SA1558	
CHORS	SA1299	
CLPNS_SA	SA1155, SA1891	yes
CO2t		
CPPPGO	SA1412	
CSNt2		
CYSS	SA0418, SA0471	yes
CYSTL	SA0346	
CYTBD	SA0910, SA0911, SA0912, SA0913, SA0937, SA0938	no
CYTD	SA1397	
CYTK1	SA1309	
DAPDC	SA0119, SA1232	yes
DAPE		
DASYN_SA	SA1104	
DB4PS	SA1587	
DDPA	SA1558	
DGK1	SA1052	

DHAD1	SA1858	
DHAD2	SA1858	
DHDPRy	SA1228	
DHDPS	SA1227	
DHFR	SA1259	
DHFS	SA1487	
DHNPA2	SA0473	
DHPPDA	SA1589	
DHPS2	SA0472	
DHQD	SA0756	
DHQS	SA1298	
DMATT	SA1352	
DPMVD	SA0548	
FCLT	SA1651	
FE2abc	SA2337, SA2369	yes
FMNAT	SA1115	
FUM	SA1669	
G1PACT	SA0457	
G1SATi	SA1491, SA1681	yes
G3PCT	SA0597	
G3PD2	SA1306	
GALUi	SA2288	
GARFT	SA0924	
GCALDD	SA2406	
GF6PTA	SA1959	
GK1	SA1052	
GLNS	SA1150	
GLUPRT	SA0922	
GLUR	SA0997	
GLUTRR	SA1496	
GLUTRS	SA0486	
GMPS2	SA0376	
GRTT	SA1352	
GTPCI	SA0683	
GTPCII	SA1587	
HCO3E	SA2287	
HEMEOS	SA0965	
HISTD	SA2470	
HISTP		
HMBS	SA1494	
HMGCOARi	SA2333	
HMGCOASi	SA2334	
HPPK2	SA0474	
HSDy	SA1164	
HSK	SA1166	
HSTPT	SA0679, SA2469	yes
IG3PS	SA2465, SA2467	no
IGPDH	SA2468	
IGPS	SA1202	
ILETA	SA0512	

IMPC	SA0925	
IMPD	SA0375	
IPDDI	SA2136	
IPMD	SA1863	
IPPMIa	SA1864, SA1865	no
IPPMIb	SA1864, SA1865	no
IPPS	SA0837, SA1862	yes
KARA1i	SA1861	
KARA2i	SA1861	
KAS1	SA0842, SA0843	no
KAS11	SA0842, SA0843	no
KAS12	SA0842, SA0843	no
KAS13	SA0842, SA0843	no
KAS19SA	SA0842, SA0843	no
KAS2	SA0842, SA0843	no
KAS20SA	SA0842, SA0843	no
KAS3	SA0842, SA0843	no
KAS4	SA0842, SA0843	no
KAS6	SA0842, SA0843	no
KAS8	SA0842, SA0843	no
LEUTA	SA0512	
MDRPD		
METAT	SA1608	
METS	SA0344, SA0345	no
MTAN	SA1427	
MTHFR2		
MTRI		
MTRK		
NACUP		
NADK	SA0865	
NADS1	SA1728	
NAPRTr	SA1729	
NDPK1	SA1301	
NDPK2	SA1301	
NDPK3	SA1301	
NDPK8	SA1301	
NNATr	SA1422	
O2t5i		
OCBT	SA1012, SA2427	yes
OIVD1	SA1346, SA1347, SA1348	no
OIVD2	SA1346, SA1347, SA1348	no
OIVD3	SA1346, SA1347, SA1348	no
OMCDC	SA1863	
ORNTAC	SA0177	
P5CD	SA2341	
PALASA_SA2		
PAPA_SA		
PAPSR		
PASYN_SA		
PDHcr	SA0946, SA1349	yes

PGAMT	SA1965	
PGCD	SA1545	
PGLYSA_SA2		
PGMT	SA1965	
PGPP_SA	SA1250	
PGSA_SA	SA1126	
PHETA1	SA0679, SA2469	yes
PLEUSA_SA2		
PLYSSA_SA2		
PMDPHT	SA1586, SA1588	no
PMEVK	SA0549	
PPA	SA1735	
PPBNGS	SA1492	
PPM	SA0134	
PPND	SA1197	
PPNDH	SA1731	
PPPGO	SA1650	
PRAGS	SA0926	
PRAI	SA1203	
PRAIS	SA0923	
PRAMPC	SA2464	
PRASCS	SA0918	
PRATPP	SA2464	
PRFGS	SA0919, SA0920, SA0921	no
PRMICIi	SA2466	
PROD2	SA1585	
PRPPS	SA0458	
PSCVT	SA1297	
PSD_SA		
PSERT		
PSP_L		
PSSA_SA		
PYNP1	SA1938	
RBFK	SA1115	
RBFSa	SA1588	
RBFSb	SA1586	
RPE	SA1065	
RPI	SA2127	
SADT2	SA0506	
SDPDS	SA1572, SA1814	yes
SDPTA	SA2347	
SERAT	SA0487	
SHCHD2	SA2412	
SHCHF	SA2412	
SHK3Dr	SA1424	
SHKK	SA1368	
SHSL1	SA0347	
SHSL4r	SA0419	
SPMS		
SUCD1	SA0994, SA0995, SA0996	no

SUCD4	SA0994, SA0995, SA0996	no
SUCOAS	SA1088, SA1089	no
SULR	SA2413	
SULabc		
TAGO		
TECA1S	SA0243, SA0244, SA0522, SA0523, SA0592, SA0593, SA0594, SA0595, SA0596, SA0597	partial
TECA2S	SA0243, SA0244, SA0522, SA0523, SA0592, SA0593, SA0594, SA0595, SA0596, SA0597, SA0793, SA0794, SA0795, SA0796	partial
TECA3S	SA0243, SA0244, SA0522, SA0523, SA0592, SA0593, SA0594, SA0595, SA0596, SA0597	partial
TECA4S	SA0243, SA0244, SA0522, SA0523, SA0592, SA0593, SA0594, SA0595, SA0596, SA0597	partial
THDPS	SA1229	
THMabc		
THRD_L	SA1271, SA1866	yes
THRS	SA1165	
TKT2	SA1177	
TMDS	SA0311, SA1260	yes
TRDR	SA0719, SA2162	yes
TRPS1	SA1204, SA1205	no
TYRTA	SA0679, SA2469	yes
UAG2E	SA0150, SA0159, SA1913	yes
UAG4E		
UAGCVT	SA1902, SA1926	yes
UAGDP	SA0457, SA1974	yes
UAPGR	SA0693	
UDPG12dgrGT_SA2		
UDPG3g12dgrGT_SA2		
UMPK	SA1309	
UNK3		
UPP3MT	SA2186	
UPP3S	SA1493	
UPPDC1	SA1652	
VALTA	SA0512	
