# Supplementary material for: Genome-scale reconstruction of the metabolic network in Staphylococcus aureus N315: an initial draft to the two-dimensional annotation
Source: BMC Microbiol. 2005 Mar 7;5:8. doi: 10.1186/1471-2180-5-8 (PMC1079855; doi:10.1186/1471-2180-5-8)
Supplement: Additional File 7 — Lethal reaction deletions on rich media This is a listing of all of the reactions that are predicted to be essential for growth on rich media and their corresponding gene associations. [file 1471-2180-5-8-S7.pdf]

Reaction Abbreviation	Gene Association	Isozymes?
ACCOAC	SA1357, SA1358, SA1434, SA1435, SA1522, SA1523	no
ADCL	SA0670	
ADCS	SA0669	
ADK1	SA2027	
ADKd	SA2027	
ADMDCr		
ADPT	SA1461	
APRAUR	SA1586, SA1588	no
ASAD	SA1226	
ASNS1	SA0922	
ASPKi	SA1163, SA1225	yes
CHORS	SA1299	
CLPNS_SA	SA1155, SA1891	yes
CYTK1	SA1309	
DASYN_SA	SA1104	
DB4PS	SA1587	
DDPA	SA1558	
DGK1	SA1052	
DHDPRy	SA1228	
DHDPS	SA1227	
DHFR	SA1259	
DHFS	SA1487	
DHNPA2	SA0473	
DHPPDA	SA1589	
DHPS2	SA0472	
DHQD	SA0756	
DHQS	SA1298	
DMATT	SA1352	
DPMVD	SA0548	
FE2abc	SA2337, SA2369	yes
FMNAT	SA1115	
G1PACT	SA0457	
G1SATi	SA1491, SA1681	yes
G3PCT	SA0597	
G3PD2	SA1306	
GALUi	SA2288	
GCALDD	SA2406	
GF6PTA	SA1959	
GK1	SA1052	
GLNS	SA1150	
GLUTRR	SA1496	
GLUTRS	SA0486	
GRTT	SA1352	
GTPCI	SA0683	
GTPCII	SA1587	
HCO3E	SA2287	
HEMEOS	SA0965	

HMBS	SA1494	
HMGCOARi	SA2333	
HMGCOASi	SA2334	
HPPK2	SA0474	
IPDDI	SA2136	
KAS1	SA0842, SA0843	no
KAS11	SA0842, SA0843	no
KAS12	SA0842, SA0843	no
KAS13	SA0842, SA0843	no
KAS19SA	SA0842, SA0843	no
KAS2	SA0842, SA0843	no
KAS20SA	SA0842, SA0843	no
KAS3	SA0842, SA0843	no
KAS4	SA0842, SA0843	no
KAS6	SA0842, SA0843	no
KAS8	SA0842, SA0843	no
MDRPD		
METAT	SA1608	
MTAN	SA1427	
MTRI		
MTRK		
NACUP		
NADK	SA0865	
NADS1	SA1728	
NAPRTr	SA1729	
NDPK1	SA1301	
NDPK2	SA1301	
NDPK3	SA1301	
NDPK8	SA1301	
NNATr	SA1422	
OIVD1	SA1346, SA1347, SA1348	no
OIVD2	SA1346, SA1347, SA1348	no
OIVD3	SA1346, SA1347, SA1348	no
PALASA_SA2		
PAPA_SA		
PASYN_SA		
PGAMT	SA1965	
PGLYSA_SA2		
PGMT	SA1965	
PGPP_SA	SA1250	
PGSA_SA	SA1126	
PLEUSA_SA2		
PLYSSA_SA2		
PMDPHT	SA1586, SA1588	no
PMEVK	SA0549	
PPA	SA1735	
PPBNGS	SA1492	
PRPPS	SA0458	

PSCVT	SA1297	
PSD_SA		
PSSA_SA		
RBFK	SA1115	
RBFSa	SA1588	
RBFSb	SA1586	
RPE	SA1065	
RPI	SA2127	
SDPDS	SA1572, SA1814	yes
SDPTA	SA2347	
SHCHD2	SA2412	
SHCHF	SA2412	
SHK3Dr	SA1424	
SHKK	SA1368	
SPMS		
TAGO		
TECA1S	SA0243, SA0244, SA0522, SA0523, SA0592, SA0593, SA0594, SA0595, SA0596, SA0597	partial
TECA2S	SA0243, SA0244, SA0522, SA0523, SA0592, SA0593, SA0594, SA0595, SA0596, SA0597, SA0793, SA0794, SA0795, SA0796	partial
TECA3S	SA0243, SA0244, SA0522, SA0523, SA0592, SA0593, SA0594, SA0595, SA0596, SA0597	partial
TECA4S	SA0243, SA0244, SA0522, SA0523, SA0592, SA0593, SA0594, SA0595, SA0596, SA0597	partial
THDPS	SA1229	
THMabc		
TKT2	SA1177	
TRDR	SA0719, SA2162	yes
UAG2E	SA0150, SA0159, SA1913	yes
UAG4E		
UAGCVT	SA1902, SA1926	yes
UAGDP	SA0457, SA1974	yes
UAPGR	SA0693	
UDPG12dgrGT_SA2		
UDPG3g12dgrGT_SA2		
UMPK	SA1309	
UNK3		
UPP3MT	SA2186	
UPP3S	SA1493	
