# Supplementary material for: Genome-scale reconstruction of the metabolic network in Staphylococcus aureus N315: an initial draft to the two-dimensional annotation
Source: BMC Microbiol. 2005 Mar 7;5:8. doi: 10.1186/1471-2180-5-8 (PMC1079855; doi:10.1186/1471-2180-5-8)
Supplement: Additional File 8 — Boolean gene-reaction associations This is a listing of reaction abbreviations along with the genes that are required for those reactions, in a Boolean form. [file 1471-2180-5-8-S8.pdf]

ABBREVIATION	NAME	ORF
3M2OBLOXRD	3-Methyl-2-oxobutanoate:lipoamide oxidoreductase(decarboxylating and acceptor-2-methylpropanoylating)	(SA1348 and SA1347)
3M2OPLOXRD	3-Methyl-2-oxopentanoate:lipoamide oxidoreductase(decarboxylating and acceptor-2-methylpropanoylating)	(SA1348 and SA1347)
4M2OPLOXRD	4-Methyl-2-oxopentanoate:lipoamide oxidoreductase(decarboxylating and acceptor-2-methylpropanoylating)	(SA1348 and SA1347)
6PGALSZ	6-phospho-beta-galactosidase	SA1991
6PHBG	6-phospho-beta-glucosidase	SA0256
ABTAr	4-aminobutyrate transaminase	SA2397
ACACT1r	acetyl-CoA C-acetyltransferase	(SA0342 or (SA0223 or SA0534))
ACACT2r	acetyl-CoA C-acyltransferase (butanoyl-CoA) (r)	(SA0342 or (SA0223 or SA0534))
ACACT3r	acetyl-CoA C-acyltransferase (hexanoyl-CoA) (r)	(SA0223 or SA0534)
ACACT4r	acetyl-CoA C-acyltransferase (octanoyl-CoA) (r)	(SA0223 or SA0534)
ACACT5r	acetyl-CoA C-acyltransferase (decanoyl-CoA) (r)	(SA0223 or SA0534)
ACACT6r	acetyl-CoA C-acyltransferase (dodecanoyl-CoA) (r)	(SA0223 or SA0534)
ACACT7r	acetyl-CoA C-acyltransferase (tetradecanoyl-CoA) (r)	(SA0223 or SA0534)
ACALDi	acetaldehyde dehydrogenase (acetylating)	SA0143
ACCOAC	acetyl-CoA carboxylase	(SA1522 and (SA1435 or SA1358) and (SA1357 or SA1434) and SA1523)
ACGApts	N-Acetyl-D-glucosamine transport via PEP:Pyr PTS	SA1547
ACGK	acetylglutamate kinase	SA0176
ACGS	N-acetylglutamate synthase	SA0177
ACHBS	2-aceto-2-hydroxybutanoate synthase	((SA1859 or SA2008) and SA1860)
ACKr	acetate kinase	SA1533
ACLDC	acetolactate decarboxylase	(SA2394 or SA2007)
ACLS	acetolactate synthase	((SA1859 or SA2008) and SA1860)
ACNAMt2	N-acetylneuraminate proton symport	SA0531
ACNML	N-Acetylneuraminate lyase	SA0304
ACOAD1	acyl-CoA dehydrogenase (butanoyl-CoA)	SA2080
ACOAD2	acyl-CoA dehydrogenase (hexanoyl-CoA)	SA2080
ACOAD3	acyl-CoA dehydrogenase (octanoyl-CoA)	SA2080
ACOAD4	acyl-CoA dehydrogenase (decanoyl-CoA)	SA2080
ACOAD5	acyl-CoA dehydrogenase (dodecanoyl-CoA)	SA2080
ACOAD6	acyl-CoA dehydrogenase (tetradecanoyl-CoA)	SA2080
ACOAD7	acyl-CoA dehydrogenase (hexadecanoyl-CoA)	SA2080
ACOATA	Acetyl-CoA ACP transacylase	SA0842
ACONT	aconitase	SA1184
ACOTA	acetylornithine transaminase	SA0179
ACPS1	acyl-carrier protein synthase	SA1875
ACPpds	[acyl-carrier-protein] phosphodiesterase	SA0204
ACS	acetyl-CoA synthetase	(SA2402 and SA1554)
ACYP_2	acylphosphatase (2)	SA1236
ADCL	4-aminobenzoate synthase	SA0670
ADCS	4-amino-4-deoxychorismate synthase	SA0669
ADCYRS	adenosylcobyric acid synthase (glutamine-hydrolysing)	SA1707
ADK1	adenylate kinase	SA2027
ADKd	adenylate kinase (d form)	SA2027
ADMDCr	Adenosylmethionine decarboxylase	
ADNt2	adenosine transport in via proton symport	
ADPRDP	ADPribose diphosphatase	SA1330
ADPT	adenine phosphoribosyltransferase	SA1461
ADSK	adenylyl-sulfate kinase	SA2456
ADSL1	adenylosuccinate lyase	SA1724
ADSL2r	adenylosuccinate lyase	SA1724

ADSS	adenylosuccinate synthase	SA0016
AGDC_r	N-acetylglucosamine-6-phosphate deacetylase (reversible)	SA0656
AGMHE	ADP-D-glycero-D-manno-heptose epimerase	SA0123
AGMT	agmatinase	SA1968
AGPR	N-acetyl-g-glutamyl-phosphate reductase	SA0178
AHCYSNS	S-adenosylhomocysteine nucleosidase	SA1427
AICART	phosphoribosylaminoimidazolecarboxamide formyltransferase	SA0925
AIRC2	phosphoribosylaminoimidazole carboxylase	SA0917
AIRC3	phosphoribosylaminoimidazole carboxylase (mutase rxn)	SA0916
AKGDa	oxoglutarate dehydrogenase (lipoamide)	SA1245
AKGDb	oxoglutarate dehydrogenase (dihydrolipoamide S-succinyltransferase)	SA1244
AKGMAL	alpha-ketoglutarate/malate transporter	SA2486
AKP1	alkaline phosphatase (Dihydroneopterin)	SA2420
ALAALAr	D-alanine-D-alanine ligase (reversible)	SA1887
ALAD_L	L-alanine dehydrogenase	(SA1272 or SA1531)
ALAR	alanine racemase	(SA1874 or SA1231)
ALATA_D	D-alanine transaminase	SA1571
ALATRS	Alanyl-tRNA synthetase	SA1446
ALAabc	L-alanine transport via ABC system	(SA1519 and SA2226 and SA2227)
ALAt2r	L-alanine reversible transport via proton symport	(SA1190 or SA0871)
ALCD1	alcohol dehydrogenase (methanol)	SA1170
ALCD2x	alcohol dehydrogenase (ethanol)	SA0562
ALDD2x	aldehyde dehydrogenase (acetaldehyde, NAD)	(SA0162 and SA1736)
ALKP	alkaline phosphatase	SA2420
AMAA	N-acetylmuramoyl-L-alanine amidase	(SA1458 or SA0905 or SA2100 or SA2437)
AMANAPEr	N-acetylmannosamine 6-phosphate epimerase	SA0307
AMANK	N-acetyl-D-mannosamine kinase	SA0715
AMAOTr	adenosylmethionine-8-amino-7-oxononanoate transaminase	SA2214
ANPRT	anthranilate phosphoribosyltransferase	SA1201
ANS	anthranilate synthase	(SA1199 and (SA1200 or SA0668))
AOXSr	8-amino-7-oxononanoate synthase	SA2212
APRAUR	5-amino-6-(5-phosphoribosylamino)uracil reductase	(SA1588 and SA1586)
ARGDC	arginine decarboxylase	SA0439
ARGDr	arginine deiminase	SA2428
ARGN	arginase	SA1968
ARGORNt7	arginine/ornithine antiporter	SA2426
ARGSL	argininosuccinate lyase	SA0821
ARGSSr	argininosuccinate synthase, reversible	SA0822
ARGTRS	Arginyl-tRNA synthetase	SA0564
ASAD	aspartate-semialdehyde dehydrogenase	SA1226
ASNN	L-asparaginase	SA1310
ASNS1	asparagine synthase (glutamine-hydrolysing)	SA0922
ASNTRS	Asparaginyl-tRNA synthetase	SA1287
ASP1DC	aspartate 1-decarboxylase	SA2390
ASPCT	aspartate carbamoyltransferase	SA1043
ASPKi	aspartate kinase, irreversible	(SA1163 or SA1225)
ASPTA	aspartate transaminase	SA1749
ASPTRS	Aspartyl-tRNA synthetase	SA1456
ASPabc	L-aspartate transport via ABC system	
ATPM	ATP maintenance requirement	
ATPPRT	ATP phosphoribosyltransferase	SA2471
ATPS4r	ATP synthase (four protons for one ATP)	(SA1907 and SA1911 and SA1904 and SA1905 and SA1909 and SA1906 and SA1908 and SA1910 and SA1912)
BACCL	biotin-[acetyl-CoA-carboxylase] ligase	SA1289

BETALDHx	betaine-aldehyde dehydrogenase	SA2406
BETALDHy	betaine-aldehyde dehydrogenase	SA2406
BPNT	3',5'-bisphosphate nucleotidase	
BTNt2i	Biotin uptake	
BTS3r	biotin synthase	SA2213
CAT	catalase	SA1170
CBMK	Carbamate kinase	(SA1013 or SA2425)
CBPS	carbamoyl-phosphate synthase (glutamine-hydrolysing)	(SA1045 and SA1046)
CDAPPA_SA	CDP-Diacylglycerol pyrophostatase (Saureus)	
CDPMEK	4-(cytidine 5'-diphospho)-2-C-methyl-D-erythritol kinase	SA0453
CHCOAL	6-carboxyhexanoate-CoA ligase	SA2211
CHLabc	choline transport via ABC system	((SA2237 and SA2236 and SA2235 and SA2234) or (SA0678 and SA0677))
CHLt2r	choline transport via proton symport, reversible	SA2408
CHOLD	choline dehydrogenase	SA2405
CHORM	chorismate mutase	SA1558
CHORS	chorismate synthase	SA1299
CIT-Mgt	citrate-Mg transport in via proton symport	SA2411
CLPNS_SA	Cardiolipin Synthase (Saureus)	(SA1891 or SA1155)
CO2t	CO2 transporter via diffusion	
CPPPGO	coproporphyrinogen oxidase (O2 required)	SA1412
CRNabc	L-carnitine transport via ABC system	((SA2237 and SA2236 and SA2235 and SA2234) or (SA0678 and SA0677))
CS	citrate synthase	SA1518
CSNt2	cytosine transport in via proton symport	
CTPS1	CTP synthase (NH3)	SA1929
CYSS	cysteine synthase	(SA0471 or SA0418)
CYSTL	cystathionine b-lyase	SA0346
CYSTRS	Cysteinyl-tRNA synthetase	SA0488
CYSabc	L-cysteine transport via ABC system	
CYTBD	cytochrome oxidase bd (ubiquinol-8: 2 protons)	(SA0937 and SA0938 and SA0913 and SA0912 and SA0911 and SA0910)
CYTD	cytidine deaminase	SA1397
CYTDK2	cytidine kinase (GTP)	SA1439
CYTDt4	cytidine transport in via sodium symport	(SA0302 or SA0479 or SA0600)
CYTK1	cytidylate kinase (CMP)	SA1309
CYTK2	cytidylate kinase (dCMP)	SA1309
Coabc	Cobalt transport via ABC system	((SA0928 and SA0927) or (SA2020 and SA2021 and SA2019) or (SA2476 and SA2475))
Cut1	Copper export via ATPase	SA2344
D-LACt2	D-lactate transport via proton symport	
DAGK_SA	Diacylglycerol kinase	SA1398
DALAt2r	D-alanine transport via proton symport	(SA1190 or SA0871)
DAPDC	diaminopimelate decarboxylase	(SA1232 or SA0119)
DAPE	diaminopimelate epimerase	
DASYN_SA	CDP-Diacylglycerol synthetase (Saureus)	SA1104
DB4PS	3,4-Dihydroxy-2-butanone-4-phosphate	SA1587
DBTSr	dethiobiotin synthase	SA2215
DCMPDA	dCMP deaminase	SA1417
DDPA	3-deoxy-D-arabino-heptulosonate 7-phosphate synthetase	SA1558
DGK1	deoxyguanylate kinase (dGMP:ATP)	SA1052
DHAD1	dihydroxy-acid dehydratase (2,3-dihydroxy-3-methylbutanoate)	SA1858
DHAD2	Dihydroxy-acid dehydratase (2,3-dihydroxy-3-methylpentanoate)	SA1858
DHAK	dihydroxyacetone kinase	SA0605
DHDPRy	dihydrodipicolinate reductase (NADPH)	SA1228
DHDPS	dihydrodipicolinate synthase	SA1227
DHFR	dihydrofolate reductase	SA1259

DHFS	dihydrofolate synthase	SA1487
DHNAOT	1,4-dihydroxy-2-naphthoate octaprenyltransferase	SA0894
DHNPA2	dihydroneopterin aldolase	SA0473
DHORD5	dihydroorotic acid (menaquinone-8)	SA2375
DHORTS	dihydroorotase	SA1044
DHPPDA	diaminohydroxyphosphoribosylaminopyrimidine deaminase	SA1589
DHPS2	dihydropteroate synthase	SA0472
DHQD	3-dehydroquinate dehydratase	SA0756
DHQS	3-dehydroquinate synthase	SA1298
DKMPPD	2,3-diketo-5-methylthio-1-phosphopentane degradation reaction	
DKMPPD2	2,3-diketo-5-methylthio-1-phosphopentane degradation reaction	
DMATT	dimethylallyltranstransferase	SA1352
DNMPPA	Dihydroneopterin monophosphate dephosphorylase	SA1313
DNTPPA	Dihydroneopterin triphosphate pyrophosphatase	SA1313
DPCOAK	dephospho-CoA kinase	SA1511
DPMVD	diphosphomevalonate decarboxylase	SA0548
DPR	2-dehydropantoate 2-reductase	(SA2232 or SA2393)
DRBK	Deoxyribokinase	SA0258
DRPA	deoxyribose-phosphate aldolase	(SA0133 or SA1939)
DTMPK	dTMP kinase	SA0440
DURIK1	deoxyuridine kinase (ATP:Deoxyuridine)	SA1921
DURIPP	deoxyuridine phosphorylase	(SA1940 or SA0131)
ECOAH1	3-hydroxyacyl-CoA dehydratase (3-hydroxybutanoyl-CoA)	SA0224
ECOAH2	3-hydroxyacyl-CoA dehydratase (3-hydroxyhexanoyl-CoA)	SA0224
ECOAH3	3-hydroxyacyl-CoA dehydratase (3-hydroxyoctanoyl-CoA)	SA0224
ECOAH4	3-hydroxyacyl-CoA dehydratase (3-hydroxydecanoyl-CoA)	SA0224
ECOAH5	3-hydroxyacyl-CoA dehydratase (3-hydroxydodecanoyl-CoA)	SA0224
ECOAH6	3-hydroxyacyl-CoA dehydratase (3-hydroxytetradecanoyl-CoA)	SA0224
ECOAH7	3-hydroxyacyl-CoA dehydratase (3-hydroxyhexadecanoyl-CoA)	SA0224
ENO	enolase	SA0731
ETOHt	ethanol reversible transport	
FACOAL160	fatty-acid--CoA ligase (hexadecanoate)	SA0226
FBA	fructose-bisphosphate aldolase	(SA1927 or SA2399)
FBP	fructose-bisphosphatase	SA2304
FCLT	Ferrochelatase	SA1651
FDHr	formate dehydrogenase	(SA0171 and SA2102)
FE2abc	iron (II) transport via ABC system	(SA2337 or SA2369)
FFSDr	beta-fructofuranosidase	SA1846
FGLU	formimidoylglutamase	SA2125
FLDO	NAD(P)H-flavin oxidoreductase	(SA0328 or SA0518)
FLVR	flavin reductase	SA2311
FLVR(NAD)	flavin reductase (NAD)	SA2311
FMNAT	FMN adenylyltransferase	SA1115
FMNRx	FMN reductase	SA0817
FORt2	formate transport in via proton symport	SA0293
FORt3	formate transport out via proton antiport	SA0293
FRUK	fructose-1-phosphate kinase	SA0654
FRUpts	D-fructose transport via PEP:Pyr PTS	SA0655
FTHFL	formate-tetrahydrofolate ligase	SA0915
FUM	fumarase	SA1669
G1PACT	glucosamine-1-phosphate N-acetyltransferase	SA0457
G1SATi	glutamate-1-semialdehyde aminotransferase	(SA1491 or SA1681)
G3PCT	glycerol-3-phosphate cytidylyltransferase	SA0597
G3PD2	glycerol-3-phosphate dehydrogenase (NADP)	SA1306

G3PD5	glycerol-3-phosphate dehydrogenase (ubiquinone-8)	SA1142
G3PD6	glycerol-3-phosphate dehydrogenase (menaquinone-8)	SA1142
G3PD7	glycerol-3-phosphate dehydrogenase (demethylmenaquinone-8)	SA1142
G6PDA	glucosamine-6-phosphate deaminase	SA0527
G6PDH2r	glucose 6-phosphate dehydrogenase	SA1336
GAL6PI	galactose-6-phosphate isomerase	(SA1997 and SA1996)
GALTpts	Galactitol transport via PEP:Pyr PTS	(SA0236 and SA0237 and SA0238)
GALUi	UTP-glucose-1-phosphate uridylyltransferase (irreversible)	SA2288
GAPD	glyceraldehyde-3-phosphate dehydrogenase	(SA0727 or SA1510)
GARFT	phosphoribosylglycinamide formyltransferase	SA0924
GCALDD	Glycolaldehyde dehydrogenase	SA2406
GCCa	glycine-cleavage complex	(SA1365 and SA1366)
GCCb	glycine cleavage complex	(SA0760 and SA1367)
GCCc	glycine-cleavage complex	(SA1349 or SA0946)
GF6PTA	glutamine-fructose-6-phosphate transaminase	SA1959
GHMT2	glycine hydroxymethyltransferase	SA1915
GK1	guanylate kinase (GMP:ATP)	SA1052
GLCNt2r	D-gluconate transport via proton symport, reversible	SA2293
GLCPcom_SA	Glucosyl Phosphoglycerol Combination (SA)	
GLCS1	glycogen synthase (ADPGlc)	SA1291
GLCpts	D-glucose transport via PEP:Pyr PTS	(SA0183 and SA1255 and (SA2326 or SA0233))
GLGC	glucose-1-phosphate adenylyltransferase	SA2288
GLNS	glutamine synthetase	SA1150
GLUDx	glutamate dehydrogenase (NAD)	SA0819
GLUPRT	glutamine phosphoribosyldiphosphate amidotransferase	SA0922
GLUR	glutamate racemase	SA0997
GLUSx	glutamate synthase (NADH2)	((SA0430 or SA2248) and SA0431)
GLUTRR	glutamyl-tRNA reductase	SA1496
GLUTRS	Glutamyl-tRNA synthetase	SA0486
GLUabc	L-glutamate transport via ABC system	(SA1674 and SA1675)
GLUt2	L-glutamate transport in via proton symport	SA2172
GLUt2r	L-glutamate transport via proton symport, reversible	(SA2172 or SA2135)
GLYALDt	Glyceraldehyde facilitated diffusion	SA1140
GLYAT	glycine C-acetyltransferase	SA0508
GLYBabc	Glycine betaine transport via ABC system	((SA2237 and SA2236 and SA2235 and SA2234) or (SA0678 and SA0677))
GLYBt2r	Glycine betaine transport via proton symport, reversible	(SA1987 or SA1183)
GLYC3Pabc	sn-Glycerol 3-phosphate transport via ABC system	(SA0207 and SA0208 and SA0209 and SA0206)
GLYC3Pt6	Glycerol-3-phosphate : phosphate antiporter	SA0325
GLYCK	glycerate kinase	(SA2220 or SA0697)
GLYCt	glycerol transport via channel	SA1140
GLYD	glycerate dehydrogenase	SA2098
GLYKr	glycerol kinase (reversible)	SA1141
GLYTRS	Glycyl-tRNA synthetase	SA1394
GLYabc	glycine transport via ABC system	(SA1519 and SA2226 and SA2227)
GMHEPAT	D-glycero-D-manno-hepose 1-phosphate adenyltransferase	SA0597
GMHEPK	D-glycero-D-manno-heptose 7-phosphate kinase	SA0597
GMHEPPA	D-glycero-D-manno-heptose 1,7-bisphosphate phosphatase	SA0513
GMPR	GMP reductase	SA1172
GMPS2	GMP synthase	SA0376
GND	phosphogluconate dehydrogenase	SA1342
GNK	gluconokinase	SA2294
GPDDA1	Glycerophosphodiester phosphodiesterase (Glycerophosphocholine)	(SA0220 or SA0820 or SA0969 or SA1542 or SA0036)
GPDDA2	Glycerophosphodiester phosphodiesterase (Glycerophosphoethanolamine)	(SA0220 or SA0820 or SA0969 or SA1542 or SA0036)
GPDDA3	Glycerophosphodiester phosphodiesterase (Glycerophosphoserine)	(SA0220 or SA0820 or SA0969 or SA1542 or SA0036)

GPDDA4	Glycerophosphodiester phosphodiesterase (Glycerophosphoglycerol)	(SA0220 or SA0820 or SA0969 or SA1542 or SA0036)
GPDDA5	Glycerophosphodiester phosphodiesterase (Glycerophosphoinositol)	(SA0220 or SA0820 or SA0969 or SA1542 or SA0036)
GRTT	geranyltranstransferase	SA1352
GTHP	glutathione peridoxase	(SA2414 or SA1146)
GTPCI	GTP cyclohydrolase I	SA0683
GTPCII	GTP cyclohydrolase II	SA1587
GTPDPK	GTP diphosphokinase	(SA1460 or SA0864)
GUAPRT	guanine phosphoribosyltransferase	SA1461
GUAt2	guanine transport in via proton symport	
H2Ot	H2O transport via diffusion	
HACD1	3-hydroxyacyl-CoA dehydrogenase (acetoacetyl-CoA)	SA0224
HACD2	3-hydroxyacyl-CoA dehydrogenase (3-oxohexanoyl-CoA)	SA0224
HACD3	3-hydroxyacyl-CoA dehydrogenase (3-oxooctanoyl-CoA)	SA0224
HACD4	3-hydroxyacyl-CoA dehydrogenase (3-oxodecanoyl-CoA)	SA0224
HACD5	3-hydroxyacyl-CoA dehydrogenase (3-oxododecanoyl-CoA)	SA0224
HACD6	3-hydroxyacyl-CoA dehydrogenase (3-oxotetradecanoyl-CoA)	SA0224
HACD7	3-hydroxyacyl-CoA dehydrogenase (3-oxohexadecanoyl-CoA)	SA0224
HCO3E	HCO3 equilibration reaction	SA2287
HCYSMT	homocysteine S-methyltransferase	SA0345
HDCAt2	Hexadecanoate transport via proton symport	
HEMEAS	Heme A synthase	SA0964
HEMEOS	Heme O synthase	SA0965
HEMEti	Heme transport via ABC system	
HETZK	hydroxyethylthiazole kinase	SA1895
HEX1	hexokinase (D-glucose:ATP)	SA1377
HEX7	hexokinase (D-fructose:ATP)	SA1845
HEXTT	trans-hexaprenyltranstransferase	SA1302
HISDr	histidase_r	SA0008
HISTD	histidinol dehydrogenase	SA2470
HISTP	histidinol-phosphatase	
HISabc	L-histidine transport via ABC system	
HMBS	hydroxymethylbilane synthase	SA1494
HMGCOARi	Hydroxymethylglutaryl CoA reductase (ir)	SA2333
HMGCOASi	Hydroxymethylglutaryl CoA synthase (ir)	SA2334
HPPK2	6-hydroxymethyl-dihydropterin pyrophosphokinase	SA0474
HSDy	homoserine dehydrogenase (NADPH)	SA1164
HSERTA	homoserine O-trans-acetylase	SA0011
HSK	homoserine kinase	SA1166
HSTPT	histidinol-phosphate transaminase	(SA0679 or SA2469)
HXPRT	hypoxanthine phosphoribosyltransferase (Hypoxanthine)	SA0468
ICDHyr	isocitrate dehydrogenase (NADP)	SA1517
ICHORS	isochorismate synthase	SA0895
IG3PS	Imidazole-glycerol-3-phosphate synthase	(SA2465 and SA2467)
IGPDH	imidazoleglycerol-phosphate dehydratase	SA2468
IGPS	indole-3-glycerol-phosphate synthase	SA1202
ILETA	isoleucine transaminase	SA0512
ILETRS	Isoleucyl-tRNA synthetase	SA1036
ILEabc	L-isoleucine transport via ABC system	
ILEt2r	L-isoleucine reversible transport via proton symport	(SA0180 or SA1239)
IMPC	IMP cyclohydrolase	SA0925
IMPD	IMP dehydrogenase	SA0375
INDPYRD	Indole-3-pyruvate carboxylase	SA0182
IPDDI	isopentenyl-diphosphate D-isomerase	SA2136
IPMD	3-isopropylmalate dehydrogenase	SA1863

IPPMIa	3-isopropylmalate dehydratase	(SA1864 and SA1865)
IPPMIb	2-isopropylmalate hydratase	(SA1864 and SA1865)
IPPS	2-isopropylmalate synthase	(SA1862 or SA0837)
IZPN	imidazolonepropionase	SA2121
KARA1i	acetohydroxy acid isomeroreductase	SA1861
KARA2i	ketol-acid reductoisomerase (2-Aceto-2-hydroxybutanoate)	SA1861
KAS1	b-ketoacyl synthetase (Iso-C14:0)	(SA0842 and SA0843)
KAS10	b-ketoacyl synthetase (Anteiso-C17:1)	(SA0842 and SA0843)
KAS11	b-ketoacyl synthetase (Iso-C17:0)	(SA0842 and SA0843)
KAS12	b-ketoacyl synthetase (Anteiso-C17:0)	(SA0842 and SA0843)
KAS13	b-ketoacyl synthetase (octadecanoate)	(SA0842 and SA0843)
KAS14	b-ketoacyl synthase	(SA0842 and SA0843)
KAS15	b-ketoacyl synthase	(SA0842 and SA0843)
KAS16	3-hydroxy-myristoyl-ACP synthesis	((SA1074 or SA0122 or SA2260) and (SA0842 and SA0843))
KAS17	b-ketoacyl synthetase (n-C18:1)	(SA0842 and SA0843)
KAS19SA	b-ketoacyl synthetase (Anteiso-C19:0)	(SA0842 and SA0843)
KAS2	b-ketoacyl synthetase (n-C14:0)	(SA0842 and SA0843)
KAS20SA	b-ketoacyl synthetase (C20:0)	(SA0842 and SA0843)
KAS3	b-ketoacyl synthetase (Iso-C15:0)	(SA0842 and SA0843)
KAS4	b-ketoacyl synthetase (Anteiso-C15:0)	(SA0842 and SA0843)
KAS5	b-ketoacyl synthetase (Iso-C16:1)	(SA0842 and SA0843)
KAS6	b-ketoacyl synthetase (Iso-C16:0)	(SA0842 and SA0843)
KAS7	b-ketoacyl synthetase (n-C16:1)	(SA0842 and SA0843)
KAS8	b-ketoacyl synthetase (palmitate, n-C16:0)	(SA0842 and SA0843)
KAS9	b-ketoacyl synthetase (Iso-C17:1)	(SA0842 and SA0843)
Kabc	Potassium ABC transporter	((SA1881 or SA0068) and (SA1880 or SA0070) and (SA1879 or SA0071))
Kt2r	potassium reversible transport via proton symport	SA0939
L-LACt2r	L-lactate reversible transport via proton symport	(SA2156 or SA0106)
LACpts	Lactose transport via PEP:Pyr PTS	(SA1992 and SA1993)
LDH_D	D-lactate dehydrogenase	(SA2312 or SA2346)
LDH_L	L-lactate dehydrogenase	(SA0232 or SA2395)
LEUTA	leucine transaminase	SA0512
LEUabc	L-leucine transport via ABC system	
LEUt2r	L-leucine reversible transport via proton symport	(SA0180 or SA1239)
LYSDC	lysine decarboxylase	SA0439
LYSTRS	Lysyl-tRNA synthetase	SA0475
LYSabc	L-lysine transport via ABC system	(SA1169 or SA1505)
LYSt2r	L-lysine reversible transport via proton symport	(SA1169 or SA1505)
M1PD	mannitol-1-phosphate 5-dehydrogenase	SA1963
MACPD	Malonyl-ACP decarboxylase	SA0843
MALT	alpha-glucosidase	SA1338
MALTAT	maltose O-acetyltransferase	SA2342
MALTabc	maltose transport via ABC system	(SA0207 and SA0208 and SA0209 and SA0206)
MAN6PI	mannose-6-phosphate isomerase	(SA1945 or SA2435)
MANpts	D-mannose transport via PEP:Pyr PTS	SA2434
MCOATA	Malonyl-CoA-ACP transacylase	SA1073
MDH	malate dehydrogenase	
MDH2	Malate dehydrogenase (ubiquinone 8 as acceptor)	SA2155
MDH3	Malate dehydrogenase (menaquinone 8 as acceptor)	SA2400
MDRPD	5-Methylthio-5-deoxy-D-ribulose 1-phosphate dehydratase	
ME1_rev	malic enzyme	SA1524
MEPCT	2-C-methyl-D-erythritol 4-phosphate cytidylyltransferase	(SA0241 or SA0245)
METAT	methionine adenosyltransferase	SA1608
METS	methionine synthase	(SA0345 and SA0344)

METTRS	Methionyl-tRNA synthetase	((SA1059 or SA0909) or SA0448)
METabc	L-methionine transport via ABC system	
MEVK1	mevalonate kinase (atp)	SA0547
MEVK2	mevalonate kinase (ctp)	SA0547
MEVK3	mevalonate kinase (gtp)	SA0547
MEVK4	mevalonate kinase (utp)	SA0547
MG2abc	magnesium transport via ABC system	SA2166
MHPGLUT	5-methyltetrahydropteroyltriglutamate-homocysteine S-methyltransferase	SA0344
MI1PP	myo-inositol 1-phosphatase	SA0958
MNLpts	mannitol transport via PEP:Pyr PTS	(SA1962 and SA1960)
MNabc	manganese transport via ABC system	(SA2194 or (SA0587 and SA0589 and SA0588) or (SA1385 and SA1384))
MNt2	manganese transport in via proton symport	SA0956
MOBDabc	molybdate transport via ABC system	(SA2074 and SA2073 and SA2072)
MOHMT	3-methyl-2-oxobutanoate hydroxymethyltransferase	SA2392
MTAN	methylthioadenosine nucleosidase	SA1427
MTHFC	methenyltetrahydrofolate cyclohydrolase	SA1553
MTHFD	methylenetetrahydrofolate dehydrogenase (NADP)	SA0915
MTHFR2	5,10-methylenetetrahydrofolate reductase (NADH)	
MTRI	5-methylthioribose-1-phosphate isomerase	
MTRK	5-methylthioribose kinase	
NACUP	Nicotinic acid uptake	
NADH10	NADH dehydrogenase (menaquinone-8 & 0 protons)	(SA0411 and SA0578 and SA0799 and SA0802 and SA0813 and SA1598)
NADH5	NADH dehydrogenase (ubiquinone-8)	(SA0411 and SA0578 and SA0799 and SA0802 and SA0813 and SA1598)
NADH6	NADH dehydrogenase (ubiquinone-8 & 3.5 protons)	(SA0411 and SA0578 and SA0799 and SA0802 and SA0813 and SA1598)
NADH7	NADH dehydrogenase (menaquinone-8 & 2 protons)	(SA0411 and SA0578 and SA0799 and SA0802 and SA0813 and SA1598)
NADH8	NADH dehydrogenase (demethylmenaquinone-8 & 2.8 protons)	(SA0411 and SA0578 and SA0799 and SA0802 and SA0813 and SA1598)
NADH9	NADH dehydrogenase (demethylmenaquinone-8 & 0 protons)	(SA0411 and SA0578 and SA0799 and SA0802 and SA0813 and SA1598)
NADK	NAD kinase	SA0865
NADS1	NAD synthase (nh3)	SA1728
NALN6	NALN6	(SA0902 or SA1544)
NAPRTr	NAPRTase (rev)	SA1729
NAt3	sodium transport out via proton antiport	(SA0813 and (SA0812 or SA0579) and (SA0811 or SA0580) and (SA0810 or SA0581) and (SA0809 or SA0582) and (SA0808 or SA0583) and (SA0807 or SA0584))
NAt3_1	sodium proton antiporter (H:NA is 1:1)	(SA2117 or SA2094)
NAt3_1.5	sodium proton antiporter (H:NA is 1.5)	(SA2228 or SA0804 or SA0585)
NAt3_2	sodium proton antiporter (H:NA is 2)	(SA2228 or SA0804 or SA0585)
NBAH	N-Benzoylglycine amidohydrolase	SA1230
NCAMUP	Nicotinamide acid uptake	
NDPK1	nucleoside-diphosphate kinase (ATP:GDP)	SA1301
NDPK2	nucleoside-diphosphate kinase (ATP:UDP)	SA1301
NDPK3	nucleoside-diphosphate kinase (ATP:CDP)	SA1301
NDPK4	nucleoside-diphosphate kinase (ATP:dTDP)	SA1301
NDPK5	nucleoside-diphosphate kinase (ATP:dGDP)	SA1301
NDPK6	nucleoside-diphosphate kinase (ATP:dUDP)	SA1301
NDPK7	nucleoside-diphosphate kinase (ATP:dCDP)	SA1301
NDPK8	nucleoside-diphosphate kinase (ATP:dADP)	SA1301
NDPK9	nucleoside-diphosphate kinase (ATP:IDP)	SA1301
NH4OHDs	NH4OH dissociation	
NH4t4	ammonium transport out via K+ antiport	SA1848
NIabc	nickel transport via ABC system	(SA2489 or (SA0228 and SA0229 and SA0230))
NNAM	nicotinamidase	SA1734
NNATr	nicotinate-nucleotide adenylyltransferase	SA1422
NO2t2r	nitrite transport in via proton symport, reversible	SA0293

NO3R1	Nitrate reductase (Ubiquinol-8)	(SA2185 and SA2184 and SA2183 and SA2182)
NO3R2	Nitrate reductase (Menaquinol-8)	(SA2185 and SA2184 and SA2183 and SA2182)
NO3t7	nitrate transport in via nitrite antiport	SA2176
NOPD	D-nopaline dehydrogenase	SA2095
NPHSr	naphthoate synthase (reversible)	SA0898
NTRIRx	nitrite reductase (NADH)	(SA2188 and SA2187)
NTRIRy	nitrite reductase (NADPH)	(SA2188 and SA2187)
O2t5i	O2 transport in via diffusion	
OCBT	ornithine carbamoyltransferase	(SA2427 or SA1012)
OCTD	D-octopine dehydrogenase	SA2095
OIVD1	2-oxoisovalerate dehydrogenase (acylating; 4-methyl-2-oxopentaoate)	(SA1348 and SA1347 and SA1346)
OIVD2	2-oxoisovalerate dehydrogenase (acylating; 3-methyl-2-oxobutanoate)	(SA1348 and SA1347 and SA1346)
OIVD3	2-oxoisovalerate dehydrogenase (acylating; 3-methyl-2-oxopentanoate)	(SA1348 and SA1347 and SA1346)
OMCDC	2-Oxo-4-methyl-3-carboxypentanoate decarboxylation	SA1863
OMPDC	orotidine-5'-phosphate decarboxylase	SA1047
OOR2r	2-oxoglutarate synthase (rev)	(SA1131 and SA1132)
ORNCD	ornithine cyclodeaminase	SA0113
ORNDC	Ornithine Decarboxylase	SA0439
ORNTA	ornithine transaminase	SA0818
ORNTAC	ornithine transacetylase	SA0177
ORNabc	ornithine transport via ABC system	(SA2200 and SA2201 and SA2202)
ORPT	orotate phosphoribosyltransferase	SA1048
P5CD	1-pyrroline-5-carboxylate dehydrogenase	SA2341
P5CR	pyrroline-5-carboxylate reductase	SA1334
PALASA_SA2	Phosphatidylalanine Synthase (SA) 2	
PANTS	pantothenate synthase	SA2391
PAPA_SA	Phosphatidate phosphatase	
PAPPT3	phospho-N-acetylmuramoyl-pentapeptide-transferase (meso-2,6-diaminopimelate)	SA1025
PAPSR	phosphoadenylyl-sulfate reductase (thioredoxin)	
PASYN_SA	Phosphatidic acid synthase (Saureus)	
PC	pyruvate carboxylase	SA0963
PDH	pyruvate dehydrogenase	(SA0944 and SA0945 and SA0946)
PDHcr	Pyruvate dehydrogenase (dihydrolipoamide dehydrogenase) reversible	(SA1349 or SA0946)
PFK	phosphofructokinase	SA1521
PFK_2	Phosphofructokinase	SA1995
PFLr	pyruvate formate lyase	SA0218
PGAMT	phosphoglucosamine mutase	SA1965
PGCD	phosphoglycerate dehydrogenase	SA1545
PGI	glucose-6-phosphate isomerase	SA0823
PGK	phosphoglycerate kinase	SA0728
PGLYCP	Phosphoglycolate phosphatase	SA0513
PGLYSA_SA2	Phosphatidylglycine Synthase (SA) 2	
PGM	phosphoglycerate mutase	(SA0361 or SA0730 or SA2204)
PGMT	phosphoglucomutase	SA1965
PGPP_SA	Phosphatidylglycerol phosphate phosphatase (Saureus)	SA1250
PGSA_SA	Phosphatidylglycerol synthase (Saureus)	SA1126
PHETA1	phenylalanine transaminase	(SA0679 or SA2469)
PHEt2r	L-phenylalanine reversible transport via proton symport	
PIabc	phosphate transport via ABC system	(SA1221 and SA1219 and SA1220 and SA1218)
PIt7	phosphate transport in/out via three Na+ symporter	SA0100
PLEUSA_SA2	Phosphatidylleucine Synthase (SA) 2	
PLYSSA_SA2	Phosphatidyllysine Synthase (SA) 2	
PMANM	phosphomannomutase	SA2279

PMDPHT	pyrimidine phosphatase	(SA1588 and SA1586)
PMEVK	phosphomevalonate kinase	SA0549
PMPK	phosphomethylpyrimidine kinase	(SA0537 or SA1896)
PNTK	pantothenate kinase	SA1439
POX	pyruvate oxidase	SA2327
POX2	Pyruvate Oxidase (2)	SA2327
PPA	inorganic diphosphatase	SA1735
PPBNGS	porphobilinogen synthase	SA1492
PPCDC	phosphopantothenoylcysteine decarboxylase	SA1054
PPCK	phosphoenolpyruvate carboxykinase	SA1609
PPM	phosphopentomutase	SA0134
PPM2	phosphopentomutase 2 (deoxyribose)	SA0134
PPNCL2	phosphopantothenate-cysteine ligase	SA1054
PPND	prephenate dehydrogenase	SA1197
PPNDH	prephenate dehydratase	SA1731
PPPGO	protoporphyrinogen oxidase	SA1650
PRAGS	phosphoribosylglycinamide synthetase	SA0926
PRAI	phosphoribosylanthranilate isomerase	SA1203
PRAIS	phosphoribosylaminoimidazole synthase	SA0923
PRAMPC	phosphoribosyl-AMP cyclohydrolase	SA2464
PRASCS	phosphoribosylaminoimidazolesuccinocarboxamide synthase	SA0918
PRATPP	phosphoribosyl-ATP pyrophosphatase	SA2464
PRFGS	phosphoribosylformylglycinamidine synthase	(SA0921 and SA0920 and SA0919)
PRMICIi	1-(5-phosphoribosyl)-5-[(5-phosphoribosylamino)methylideneamino)imidazole-4-carboxamide isomerase (irreversible)	SA2466
PROD2	Proline dehydrogenase	SA1585
PROabc	L-proline transport via ABC system	((SA2237 and SA2236 and SA2235 and SA2234) or (SA0678 and SA0677))
PROt4	Na+/Proline-L symporter	SA1718
PRPPS	phosphoribosylpyrophosphate synthetase	SA0458
PSCVT	3-phosphoshikimate 1-carboxyvinyltransferase	SA1297
PSD_SA	Phosphatidylserine decarboxylase (Saureus)	
PSERT	phosphoserine transaminase	
PSP_L	phosphoserine phosphatase (L-serine)	
PSSA_SA	Phosphatidylserine syntase (Saureus)	
PTAr	phosphotransacetylase	SA0545
PTHPS	6-pyruvoyltetrahydropterin synthase	SA0666
PTPATi	pantetheine-phosphate adenylyltransferase	SA0973
PTRCORNt7	putrescine/ornithine antiporter	SA1270
PUNP1	purine-nucleoside phosphorylase (Adenosine)	(SA1940 or SA0131)
PUNP2	purine-nucleoside phosphorylase (Deoxyadenosine)	(SA1940 or SA0131)
PYK	pyruvate kinase	SA1520
PYNP1	pyrimidine-nucleoside phosphorylase (cytosine)	SA1938
PYRZAM	Pyrazinamidase	SA1734
RAFH	raffinose hydrolyzing enzyme	SA1846
RBFK	riboflavin kinase	SA1115
RBFSa	riboflavin synthase	SA1588
RBFSb	riboflavin synthase	SA1586
RBK	ribokinase	SA0258
RBK_L1	L-ribulokinase (L-ribulose)	SA0510
RIBabc	D-ribose transport via ABC system	(SA0259 and SA0260)
RNDR1	ribonucleoside-diphosphate reductase (ADP)	(SA0686 and SA0687)
RNDR2	ribonucleoside-diphosphate reductase (GDP)	(SA0686 and SA0687)
RNDR3	ribonucleoside-diphosphate reductase (CDP)	(SA0686 and SA0687)
RNDR4	ribonucleoside-diphosphate reductase (UDP)	(SA0686 and SA0687)

RNTR1	ribonucleoside-triphosphate reductase (ATP)	SA2410
RNTR2	ribonucleoside-triphosphate reductase (GTP)	SA2410
RNTR3	ribonucleoside-triphosphate reductase (CTP)	SA2410
RNTR4	ribonucleoside-triphosphate reductase (UTP)	SA2410
RPE	ribulose 5-phosphate 3-epimerase	SA1065
RPI	ribose-5-phosphate isomerase	SA2127
S3AdT	streptomycin 3''-adenylyltransferase	(SA2385 or SA1952 or SA1481 or SA0765 or SA0049)
S7PIr	sedoheptulose 7-phosphate isomerase (reversible)	SA0306
SADT2	Sulfate adenyltransferase	SA0506
SALCpts	salicin transport via PEP:Pyr PTS	SA0255
SBTD_Dr	D-sorbitol dehydrogenase (R)	(SA0239 or SA0240)
SDPDS	succinyl-diaminopimelate desuccinylase	(SA1572 or SA1814)
SDPTA	succinyldiaminopimelate transaminase	SA2347
SERAT	serine O-acetyltransferase	SA0487
SERD_Lr	L-serine deaminase (rev)	(SA2318 and SA2319)
SERTRS	Seryl-tRNA synthetase	SA0009
SERabc	L-serine transport via ABC system	(SA1519 and SA2226 and SA2227)
SHCHCS2	2-succinyl-6-hydroxy-2,4-cyclohexadiene 1-carboxylate synthase	SA0896
SHCHD2	sirohydrochlorin dehydrogenase (NAD)	SA2412
SHCHF	sirohydrochlorin ferrochetalase	SA2412
SHK3Dr	shikimate dehydrogenase	SA1424
SHKK	shikimate kinase	SA1368
SHSL1	O-succinylhomoserine lyase (L-cysteine)	SA0347
SHSL4r	O-succinylhomoserine lyase (elimination), reversible	SA0419
SPMDabc	spermidine transport via ABC system	(SA0950 and SA0951 and SA0952 and SA0953)
SPMS	spermidine synthase	
SPODM	superoxide dismutase	(SA1382 or SA0128)
SQLS	Squalene synthase	SA2348
SSALy	succinate-semialdehyde dehydrogenase (NADP)	SA1924
SUCBZL	o-succinylbenzoate-CoA ligase	SA1615
SUCBZS	O-succinylbenzoate-CoA synthase	SA1614
SUCD1	succinate dehydrogenase	(SA0995 and SA0996 and SA0994)
SUCD4	succinate dehyrdogenase	(SA0995 and SA0996 and SA0994)
SUCOAS	succinyl-CoA synthetase (ADP-forming)	(SA1088 and SA1089)
SUCR	sucrose hydrolyzing enzyme	SA1846
SUCpts	sucrose transport via PEP:Pyr PTS	SA2167
SULR	sulfite reductase (NADPH2)	SA2413
SULabc	sulfate transport via ABC system	
Spt3AdT	spectinomycin 3''-adenylyltransferase	(SA2385 or SA1952 or SA1481 or SA0765 or SA0049)
TAGO	Tag O reaction for initiation of teichoic acid	
TALA	transaldolase	SA1599
TECA1S	glycerol teichoic acid synthesis (no sub, n=25)	(SA0592 and (SA0522 or SA0523) and (SA0243 or SA0595) and SA0597 and SA0244 and SA0594 and SA0593 and SA0596)
TECA2S	glycerol techoic acid synthesis (D-ala, n=25)	(SA0793 and SA0794 and SA0795 and SA0796 and SA0592 and (SA0522 or SA0523) and (SA0243 or SA0595) and SA0597 and SA0244 and SA0594 and SA0593 and SA0596)
TECA3S	glycerol teichoic acid synthesis (n=25, glucose sub)	(SA0592 and (SA0522 or SA0523) and (SA0243 or SA0595) and SA0597 and SA0244 and SA0594 and SA0593 and SA0596)
TECA4S	minor teichoic acid synthesis (n=30)	(SA0592 and (SA0522 or SA0523) and (SA0243 or SA0595) and SA0597 and SA0244 and SA0594 and SA0593 and SA0596)
TEST_NADTRHD	NAD transhydrogenase	
TGBPA	Tagatose-bisphosphate aldolase	SA1994
THDPS	tetrahydrodipicolinate succinylase	SA1229
THFGLUS	Tetrahydrofolate:L-glutamate gamma-ligase (ADP-forming)	SA1487

THMDt2	thymidine transport in via proton symport	
THMDt4	thymidine transport in via sodium symport	(SA0302 or SA0479 or SA0600)
THMabc	thiamine transport via ABC system	
THRAr	Threonine Aldolase	SA1154
THRD_L	L-threonine deaminase	(SA1271 or SA1866)
THRS	threonine synthase	SA1165
THRTRS	Threonyl-tRNA synthetase	SA1506
THRt2	L-threonine transport in via proton symport	
TKT1	transketolase	SA1177
TKT2	transketolase	SA1177
TMDK1	thymidine kinase (ATP:thymidine)	SA1921
TMDS	thymidylate synthase	(SA1260 or SA0311)
TMPPP	thiamine-phosphate diphosphorylase	SA1894
TPI	triose-phosphate isomerase	SA0729
TRDR	thioredoxin reductase (NADPH)	(SA2162 or SA0719)
TRE6PH	trehalose-6-phosphate hydrolase	SA0433
TREpts	trehalose transport via PEP:Pyr PTS	SA0432
TRPS1	tryptophan synthase (indoleglycerol phosphate)	(SA1204 and SA1205)
TRPt2r	L-tryptophan reversible transport via proton symport	
TYRTA	tyrosine transaminase	(SA0679 or SA2469)
TYRt2r	L-tyrosine reversible transport via proton symport	
UAAGDS	UDP-N-acetylmuramoyl-L-alanyl-D-glutamyl-meso-2,6-diaminopimelate synthetase	(SA0876 or SA1708)
UAG2E	UDP-N-acetylglucosamine 2-epimerase	(SA1913 or (SA0159 or SA0150))
UAG2EMA	UDP-N-acetyl-D-glucosamine 2-epimerase (Hydrolysis)	(SA1913 or (SA0159 or SA0150))
UAG4E	UDP-N-acetylglucosamine 4-epimerase	
UAGCVT	UDP-N-acetylglucosamine 1-carboxyvinyltransferase	(SA1902 or SA1926)
UAGDP	UDP-N-acetylglucosamine diphosphorylase	(SA1974 or SA0457)
UAGPT3	UDP-N-acetylglucosamine-N-acetylmuramyl-(pentapeptide)pyrophosphoryl-undecaprenol N-acetylglucosamine transferase	SA1251
UAMAGS	UDP-N-acetylmuramoyl-L-alanyl-D-glutamate synthetase	SA1026
UAMAS	UDP-N-acetylmuramoyl-L-alanine synthetase	SA1561
UAPGR	UDP-N-acetylenolpyruvoylglucosamine reductase	SA0693
UDCPKr	undecaprenol kinase (reversible)	SA0638
UDPDPS	undecaprenyl-diphosphate synthase	SA1103
UDPG12dgrGT_SA2	UDPglucose:1,2-diacylglycerol 3-D-glucosyltransferase	
UDPG3g12dgrGT_SA2	UDPglucose:3g12dgr 3-D-glucosyltransferase 2	
UDPG4E	UDPglucose 4-epimerase	SA0123
UGMDDS	UDP-N-acetylmuramoyl-L-alanyl-D-glutamyl-meso-2,6-diaminopimeloyl-D-alanyl-D-alanine synthetase	SA1886
UMPK	UMP kinase	SA1309
UNK3	2-keto-4-methylthiobutyrate transamination	
UPP3MT	uroporphyrinogen methyltransferase	SA2186
UPP3S	uroporphyrinogen-III synthase	SA1493
UPPDC1	uroporphyrinogen decarboxylase (uroporphyrinogen III)	SA1652
UPPDC2	uroporphyrinogen decarboxylase (uroporphyrinogen I)	SA1652
UPPRTr	uracil phosphoribosyltransferase (r)	(SA1041 or SA1914)
URAt2	uracil transport in via proton symport	SA1042
URCN	urocanase	SA2122
UREA	urease	(SA2082 and SA2084 and SA2083 and SA2088 and SA2085 and SA2086 and SA2087)
UREAt	Urea transport via facilitate diffusion	(SA2081 or SA1140)
URFGTT	UDP-L-rhamnose:flavonol-3-O-D-glucoside L-rhamnosyltransferase	SA1303
URIDK2r	uridylate kinase (dUMP)	SA1101

URIK1	uridine kinase (ATP:Uridine)	SA1439
URIK2	uridine kinase (GTP:Uridine)	SA1439
URIK3	uridine kinase (ITP:Uridine)	SA1439
URIt4	uridine transport in via sodium symport	(SA0302 or SA0479 or SA0600)
VALTA	valine transaminase	SA0512
VALabc	L-valine transport via ABC system	
VALt2r	L-valine reversible transport via proton symport	(SA0180 or SA1239)
XANt	xanthine reversible transport	SA0374
XANt2	xanthine transport in via proton symport	SA0374
XPPT	xanthine phosphoribosyltransferase	SA0373
YUMPS	yUMP synthetase	(SA1114 or (SA1574 and SA2018) or SA1324)
ZNabc	zinc transport via ABC system	(SA2194 or (SA0587 and SA0589 and SA0588) or (SA1385 and SA1384))
