# Supplementary material for: Genome-scale reconstruction of the metabolic network in Staphylococcus aureus N315: an initial draft to the two-dimensional annotation
Source: BMC Microbiol. 2005 Mar 7;5:8. doi: 10.1186/1471-2180-5-8 (PMC1079855; doi:10.1186/1471-2180-5-8)
Supplement: Additional File 9 — Reactions that were added based on systemic evidence This is a list of the reactions that were added to the reconstruction based on systemic analysis (they do not appear in an obvious fashion in the genome annotation), and the gene(s) with which they are associated in cases where a gene could be located based on homology searches. [file 1471-2180-5-8-S9.pdf]

**Reaction**	**Gene**	**Best E value**	**Organism (EC or BS)**
ACNAMt2	SA0531	2.00E-19	EC
ADMDCr			
ADNt2			
ADSK	SA2456	3.80E-02	BS
AGMHE	SA0123	8.00E-17	EC
AGMT	SA1968	7.00E-23	BS
ASNS1	SA0922	6.00E-10	BS
ASPabc			
ATPM			
BPNT			
BTN2i			
CDAPPA_SA			
CO2t			
CSNt2			
CYSabc			
DAPE			
DKMPPD			
DKMPPD2			
D-LACt2			
ETOHt			
GCALDD	SA2406	2.00E-81	EC
GLCS1	SA1291	0.004	BS
GLGC	SA2288	0.001	BS
GMHEPAT	SA0597	8.00E-09	EC
GMHEPK	SA0597	8.00E-09	EC
GMHEPPA	SA0513	6.00E-17	EC
GUAt2			
H2Ot			
HCO3E	SA2287	0.046	BS
HDCAt2			
HEMEti			
HISabc			
HISTP			
ILEabc			
KDOPS	SA1558	2.00E-18	EC
LEUabc			
MACPD	SA0843	5.00E-59	EC
MDH			
MDRPD			
METabc			
MTHFR2			
MTRI			
MTRK			
NACUP			
NCAMUP			
NH4OHDs			
O2t5i			
ORNabc	SA2200	3.00E-61	EC
ORNabc	SA2201	3.00E-18	EC
ORNabc	SA2202	9.00E-18	EC

PALASA_SA2			
PAPA_SA			
PAPSR			
PASYN_SA			
PGLYSA_SA2			
PGPP_SA	SA1250	0.002	EC
PHEt2r			
PLEUSA_SA2			
PLYSSA_SA2			
PNTK	SA1439	5.00E-11	BS
PSD_SA			
PSERT			
PSP_L			
PSSA_SA			
PTRCORNt7	SA1270	2.00E-21	EC
S7PIr	SA0306	7.00E-04	EC
SADT2	SA0506	5.00E-21	EC
SADT2	SA0506	5.00E-21	EC
SPMS			
SULabc			
TAGO			
TEST_NADTRHD			
THMabc			
THMDt2			
THRt2			
TRPt2r			
TYRt2r			
U23GAAT	SA0457	6.00E-07	EC
UAG4E			
UAGAAT	SA0457	4.00E-05	EC
UDPG12dgrGT_SA2			
UDPG3g12dgrGT_SA2			
UNK3			
USHD	SA0022	4.00E-41	EC
VALabc			
